# LEAF-E: a tool to analyze grass leaf growth using function fitting

**DOI:** 10.1186/1746-4811-10-37

**Published:** 2014-11-06

**Authors:** Wannes Voorend, Peter Lootens, Hilde Nelissen, Isabel Roldán-Ruiz, Dirk Inzé, Hilde Muylle

**Affiliations:** Department of Plant Systems Biology, VIB, Technologiepark 927, 9052 Gent, Belgium; Department of Plant Biotechnology and Bioinformatics, Ghent University, Technologiepark 927, 9052 Gent, Belgium; Plant Sciences Unit – Growth and Development, Institute for Agricultural and Fisheries Research (ILVO), Caritasstraat 21, 9090 Melle, Belgium

**Keywords:** Leaf elongation rate, Non-linear regression, Leaf length, Cell length, Growth zone

## Abstract

In grasses, leaf growth is often monitored to gain insights in growth processes, biomass accumulation, regrowth after cutting, etc. To study the growth dynamics of the grass leaf, its length is measured at regular time intervals to derive the leaf elongation rate (LER) profile over time. From the LER profile, parameters such as maximal LER and leaf elongation duration (LED), which are essential for detecting inter-genotype growth differences and/or quantifying plant growth responses to changing environmental conditions, can be determined. As growth is influenced by the circadian clock and, especially in grasses, changes in environmental conditions such as temperature and evaporative demand, the LER profiles show considerable experimental variation and thus often do not follow a smooth curve. Hence it is difficult to quantify the duration and timing of growth. For these reasons, the measured data points should be fitted using a suitable mathematical function, such as the beta sigmoid function for leaf elongation.

In the context of high-throughput phenotyping, we implemented the fitting of leaf growth measurements into a user-friendly Microsoft Excel-based macro, a tool called LEAF-E. LEAF-E allows to perform non-linear regression modeling of leaf length measurements suitable for robust and automated extraction of leaf growth parameters such as LER and LED from large datasets. LEAF-E is particularly useful to quantify the timing of leaf growth, which forms an important added value for detecting differences in leaf growth development. We illustrate the broad application range of LEAF-E using published and unpublished data sets of maize, *Miscanthus* spp. and *Brachypodium distachyon*, generated in independent experiments and for different purposes. In addition, we show that LEAF-E could also be used to fit datasets of other growth-related processes that follow the sigmoidal profile, such as cell length measurements along the leaf axis.

Given its user-friendliness, ability to quantify duration and timing of leaf growth and broad application range, LEAF-E is a tool that could be routinely used to study growth processes following the sigmoidal profile.

**Electronic supplementary material:**

The online version of this article (doi:10.1186/1746-4811-10-37) contains supplementary material, which is available to authorized users.

## Background

Leaf growth has been monitored in a wide variety of grass species such as maize, rice, wheat, barley, *Lolium*, *Miscanthus, Sorghum* and *Brachypodium* making use of leaf length measurements taken at regular time intervals during development [1–8]. Parameters derived from these measurements such as leaf elongation rate (LER) and leaf elongation duration (LED) have been shown to be major determinants of individual and whole plant leaf area [9–14] and can be used to explain differences in final leaf length in response to environmental conditions and/or between genotypes [3, 4, 15].

In plant growth modeling, there is a growing consensus that approaches applying linear and exponential models are inadequate [16]. A linear fit assumes a constant LER over a longer period during leaf development [1, 3, 9, 10] and an exponential or a log-linear relation assumes a constant relative elongation rate (RER). These assumptions limit the utility of the models, as both LER and RER may vary with environmental conditions and developmental stage [16]. The polynomial model does cope with variations in LER and RER during leaf development. However, polynomial functions tend to make spurious upward or downward predictions, especially at the extremes of the data [16, 17]. Nonlinear regression is a more suitable strategy to describe leaf growth and to accommodate temporal variation in growth rates [16].

The beta sigmoid function, first used to describe whole plant growth [18], has been successfully applied to model the growth pattern of a single grass leaf [7, 19]. Yin and coworkers [18] compared the performance of the beta sigmoid function with that of some other widely used sigmoid functions, such as Gompertz, Weibull and Richards to analyze datasets from maize, pea and wheat and concluded that the beta sigmoid function is unique in dealing with determinate growth [18]. This is due to the prediction of a zero growth rate at both the start and end of the determinate growth period which is characterized by three sub-phases: an early exponential growth phase, an approximately linear growth phase, followed by a steadily decelerating growth phase [20]. Furthermore, in contrast to other functions, the beta sigmoid function incorporates biologically relevant parameters and is highly flexible for describing various asymmetrical sigmoidal patterns [18].

In the context of high-throughput leaf phenotyping, there is a need for user-friendly tools that provide rapid and robust analysis of growth parameters from large datasets. Non-linear regression using function fitting is currently imbedded in statistical work packages such as SAS and R rendering the calculation, extraction and visualization of specific leaf growth parameters, such as LED, from large datasets difficult and time-consuming.

Here, we describe LEAF-E, a nonlinear regression-based tool for analyzing grass leaf growth data. The tool can be used to derive biologically relevant parameters such as final leaf length, maximal LER, LED but also parameters for the quantification of the timing of leaf growth, an important asset of this tool. To allow for the analysis of large datasets, the fitting procedure was automated in a user-friendly Microsoft Excel macro, which is innovative. We show how the application of this tool can assist data analysis and interpretation of experiments in which different genotypes or the response of single genotypes to different growth conditions are compared. For this purpose, we quantified and compared leaf growth parameters in published and unpublished datasets of three grass species: *Zea mays* (maize), *Brachypodium distachyon* and *Miscanthus spp*.

## Results and discussion

### Fitting of kinematic individual leaf length measurements using the beta sigmoid function

#### Goodness of fit of the beta sigmoid function for maize, *Brachypodium*and *Miscanthus*leaf growth

**Figure 1:**
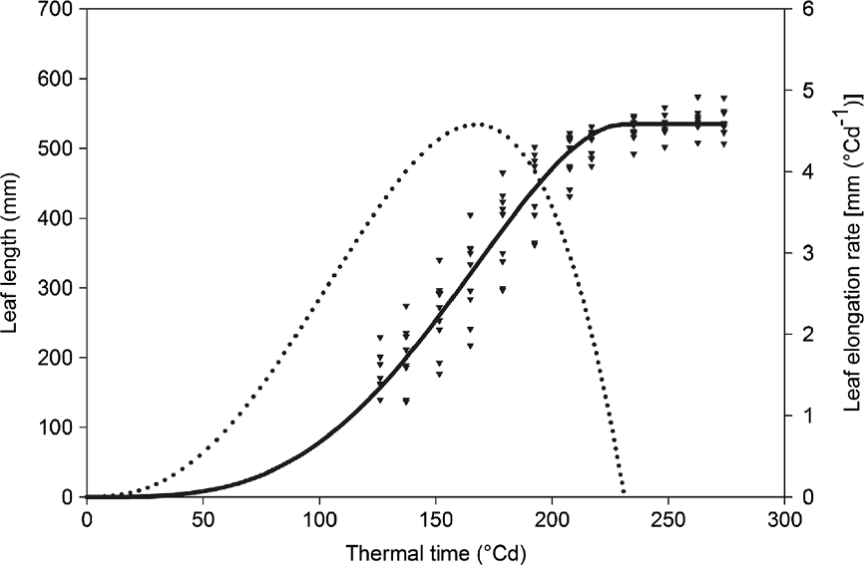
**Leaf length and LER of maize B104 non-transgenic plants.** Triangles represent length measurements of the 4^th^ leaf of nine non-transgenic maize plants from dataset1a. The measurements of each individual leaf were fitted and biologically relevant growth parameters were extracted with LEAF-E. The S-shaped leaf length curve and bell-shaped LER curve are function plots using the mean values of the function parameters for the nine non-transgenic maize plants.

First, we investigated to what extent the beta sigmoid function can be used to accurately fit leaf length measurements in function of thermal time or growing degree days (°Cd) in the three species considered here. Thermal time was used since plant growth and development are more closely related to accumulated mean daily temperature above a base value in the absence of other limiting conditions [21, 22]. Equation 1 was used to fit length measurements of the 4^th^ leaf over thermal time of nine B104 maize plants (dataset 1a, Figure 1). This resulted in R^2^ values ranging from 0.9970 to 0.9989 with a mean value of 0.9981. Function fitting of leaf length measurements in *Miscanthus* and *Brachypodium* (datasets 2 and 3, respectively) rendered similar results: an overall mean R^2^-value of 0.9931, ranging from 0.9669 to 0.9989 (n = 18) for the two *Miscanthus* species, and an overall mean R^2^-value of 0.9932, ranging from 0.9871 to 0.9993 (n = 36) for the four *Brachypodium* inbred lines. Plots of the fittings and R^2^-values of individual plants of all datasets can be found in Additional file 1. A linear regression analysis of the measured leaf lengths versus the estimated value for those respective points in thermal time resulted in an R^2^ value of 0.9986 for maize (dataset 1a), 0.9951 for *Miscanthus* (dataset 2) and 0.9940 for *Brachypodium*, indicating a good fit of the data for the three species investigated (Additional file 2 A, D and G). Nevertheless, analysis of the residuals showed a remaining sinusoidal pattern which indicates that part of the data could not be explained by the model (Additional file 3). The higher R^2^ value for the fittings of the maize dataset over the *Miscanthus* and *Brachypodium* datasets might be due to the more controlled environment of the growth chamber for maize as compared to the greenhouse for *Miscanthus* and *Brachypodium*. This indicates the importance of conducting growth experiments in a controlled environment and/or close monitoring of important growth regulating factors such as temperature, preferably at the level of the plant apex.

Based on these results, we can conclude that the beta sigmoid function is able to accommodate leaf growth measurements of three grass species with very distinct phenotypic characteristics. Maize and *Miscanthus spp.* both possess a ‘C4’ metabolism, however, maize is an annual crop characterized by one stem, whereas *Miscanthus spp.* are rhizomatous perennials that form numerous tillers. *Brachypodium* is a small, annual ‘C3’ plant used as a model for several temperate grain crops such as wheat and barley [23]. Based upon these findings and the results obtained previously in *L. perenne* [7], we can conclude that the beta sigmoid function is probably of broad application for describing leaf growth in both C3 and C4 grass species. For that reason, the beta sigmoid function was further used to develop an automated fitting procedure in an excel macro that we called LEAF-E and to derive biologically relevant parameters from the different datasets.

### Biologically relevant function parameters

The Excel macro that we designed, automatically generates the fitted function parameters (the final leaf length *Lm*, the moment of maximal leaf elongation rate *tm*, and the moment at which leaf growth ceases *te*) and all additional parameters in the form of a table. For visual inspection, it also generates a graph showing the original data points, the fitted growth curve and the function parameters for each biological replicate (see Additional files 4 and 5). Although the tool can be used to fit the measurements of several replicates jointly, fitting data of individual leaves allows statistical analysis, such as estimation of averages and standard deviation values, for all growth parameters, and comparison of genotypes or treatments, a strategy that is both straightforward and statistically correct [17]. The advantage of using the beta sigmoid function in the form of Auzanneau and coworkers [7] is that the function parameters (*Lm*, *tm* and *te*) themselves are biologically relevant when assessing grass leaf growth. This represents a clear advantage over functions that are based on parameters with no biologically relevant meaning or parameters that are difficult to interpret visually such as the Weibull equation [18]. In addition, parameters that allow for the quantification of the timing of leaf growth, such as *tm* and *te*, can be estimated, which is not trivial using other methodologies such as a log-linear, polynomial or linear fit.

We used the nine B104 maize plants from dataset 1a to illustrate how leaf growth can be analyzed using LEAF-E (Figure 1, Table 1). The final length (*Lm*) of the 4^th^ leaf in the nine B104 maize plants was 535 ± 6 mm on average. This value was attained after 231 ± 5°Cd (*te*), which, in this experiment, is equivalent to 16.5 days after sowing. The moment at which the LER was maximal, *tm*, was 167 ± 5°Cd, or 12.0 days after sowing.

**Table 1:** **Effect of** ***GA20ox1*** **overexpression on maize leaf elongation**

Growth parameter			***AtGA20ox1***OE (mean ± SE)	Control (mean ± SE)	Difference in mean ^(+)^	
	Lm	(mm)	743 ± 13	535 ± 6	38.9%	***
	LERmax	(mm°C^-1^d^-1^)	6.2 ± 0.2	4.6 ± 0.1	34.2%	***
Thermal time points	t100	(°Cd)	107 ± 2	108 ± 4	-1.7%	NS
	t20%	(°Cd)	121 ± 3	111 ± 4	8.7%	NS
	t50%	(°Cd)	165 ± 3	153 ± 5	7.6%	*
	tm	(°Cd)	180 ± 4	167 ± 5	7.7%	*
	t90%	(°Cd)	217 ± 4	203 ± 5	6.7%	*
	te	(°Cd)	246 ± 5	231 ± 5	6.3%	*
Leaf elongation durations	LED(100-e)	(°Cd)	139 ± 4	123 ± 2	13.3%	**
	LED(20%-90%)	(°Cd)	96 ± 2	92 ± 2	4.2%	NS
	LED(20%-e)	(°Cd)	125 ± 30	120 ± 2	4.0%	NS

A segregating population produced by backcrossing (BC) a transgenic plant overexpressing the *Arabidopsis thaliana GIBBERELLIC ACID 20 OXIDASE1 (GA20ox1) gene* to the wild-type line B104 was analysed for leaf growth. The results are based on the analysis of eleven transgenic and nine non-transgenic BC1 plants. Lm: final leaf length; LERmax: maximal leaf elongation rate; t20%, t50%, t90%, te: time points at which the leaf reaches 20%, 50%, 90% and 100% of the final leaf length, respectively; t100: time point at which the leaf reaches 100 mm; tm: time point at which the leaf reaches LERmax; LEDs: leaf elongation durations between above stated thermal time points.

+Statistical significance based on student t-test of non-transgenic plants (n = 9) vs *GA20ox1* overexpression (n = 11), *p < 0.05, ** p < 0.01, ***p < 0.001, NS non-significant. Applied base temperature for thermal time calculation = 10°C, Mean of overall R^2^ values = 0.9983 (0.9970-0.9991).

### Flexibility to extract additional biologically relevant information from the dataset

**Figure 2:**
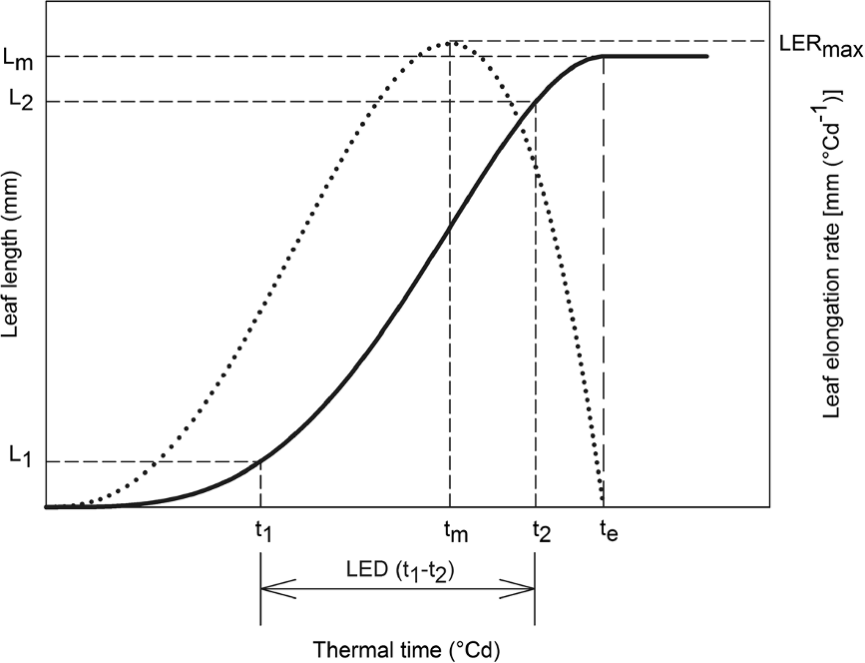
**Deriving leaf growth parameters from the fitted leaf length and LER curve using LEAF-E.** The leaf length curve (S-shaped) is generated by fitting Equation 1 to measurements of a single leaf. Based upon the leaf length curve the final leaf length *L*_*m*_ can be calculated as well as the thermal time *t* needed to reach any given leaf length L, e.g. *te* is the thermal time needed to reach *L*_*m*_. As a result, LEDs can be calculated between every desirable pair of thermal time points, e.g. LED(t1-t2). The first derivative of Equation 1 renders a bell-shaped LER curve from which maximal leaf elongation rate *LERmax*, occurring at the thermal time point *tm*, can be extracted.

The major advantage of fitting a continuous function to the data is that for any given thermal time *t* (°Cd), the leaf length *L* (mm) can be estimated and vice versa (Figure 2).

For example, the leaves of the nine B104 maize plants of dataset 1a (Table 1) reached 100 mm (*t100*) at 108 ± 4°Cd. In this experiment this was just before the 4^th^ leaf emerged from the pseudo-stem or whorl (the spiral arrangement of leaves forming a cylindrical structure from where newly formed leaves emerge), approximately 8 days after sowing. Knowing that the average final length (*Lm*) is 535 ± 6 mm, we can state that a considerable share of at least 19% of the final maize leaf length is hidden in the pseudo-stem of this genotype. For *t20%*, the moment at which the 4^th^ leaf reaches 20% of its final length, we obtained an estimate of 111 ± 4°Cd or 7.9 days after sowing. Exactly 50% of the final leaf length was attained at on average 153 ± 5°Cd (*t50%*), which is not significantly different from the moment of the maximal LER, *tm* at 167 ± 5°Cd. The leaf reached 90% of its final leaf length (*t90%*) at 203 ± 5°Cd or 14.5 days after sowing. The use of parameters that are independent of final leaf length, such as *t20%*, *t50%* and *t90%*, can however be meaningful when comparing genotypes that differ inherently in final leaf lengths. Likewise, we calculated a time window between reaching *20%* and *90%* of the final leaf length. The parameter was named [*LED(20%-90%)*] and corresponded to 92 ± 2°Cd or 6.7 days in this experiment (dataset 1a). Depending on the objective of a particular experiment, several other useful parameters can be extracted from the growth curve. Parameters can be deduced from the model, even at time points before empirical evidence can be taken (for maize e.g. t100 and in some cases also t20%) using non-destructive measurements of leaf length over time, as the leaf is then still hidden in the pseudo-stem. However, these are approximations and will need validation using destructive measurements of leaf elongation when one wants to investigate early leaf development, i.e. when the leaf is still hidden in the pseudo-stem [24, 25].

### Leaf elongation rate and steady-state growth

Similarly, for any given thermal time *t* (°Cd), the LER (mm/°Cd) can be estimated using the first derivative of the beta sigmoid function (Equation 2). The maximum of this bell-shaped curve, is denoted as the maximal LER or *LERmax* (Figure 2). For the nine B104 maize plants (dataset 1a), we estimated a *LERmax* of 4.6 ± 0.1 mm/°Cd, equivalent to an impressive 2.7 mm/h or 64 mm/day.

Often, the LER profile is derived from calculations of leaf length increases between consecutive measurements divided by the respective thermal time interval. These calculations were performed on a plant-by-plant basis and can be viewed in Additional file 1. The LERmax is then defined as the maximal value in that LER profile. A linear regression analysis of the estimated LER and LERmax to the calculated LER and LERmax, respectively showed high R² values (Additional file 2), indicating a linear relationship between values obtained by the two approaches. The R² values of the linear regression were highest for the maize dataset (dataset 1a), 0.9067 and 0.9228 for LER and LERmax respectively (see Additional file 2 for R² values of all linear regression analyses).

Several studies have assumed that during the growth of a grass leaf a period of constant LER can be defined [5, 15, 25, 26]. This assumption of steady-state growth in grass species such as maize has often been used as an acceptable simplification of the actual growth process [5, 15, 25, 26], although it has been argued that this steady-state growth period is relatively short compared to the total leaf growth period [8, 25, 27]. LEAF-E allows to quantify a steady state growth period. This can be defined as a period in leaf development for which the LER is relatively stable, for example the thermal time window between the points at which LER has a value of 90% or 95% of LERmax. For the maize B104 plants of dataset 1a, the thermal time window for which the LER was higher than 90% and 95% of LERmax was 34.5 ± 0.6°Cd or 2.5 days (from 10.7 until 13.1 days after sowing) and 49.1 ± 0.9°Cd or 3.5 days (from 10.1 until 13.6 days after sowing), respectively. We therefore conclude that, for the investigated dataset, a relatively stable LER is found for a time-span of 2.5 to 3.5 days during leaf growth.

### Effect of *GA20ox1*overexpression on maize leaf elongation

Comparison of transgenic maize plants overexpressing the GA biosynthesis gene *GA20ox1* with non-transgenic plants in a previous study, demonstrated that altering GA levels specifically affects the size of the division zone, resulting in proportional changes in leaf and whole plant growth rates [28]. This published dataset was used to validate the LEAF-E tool.

**Figure 3:**
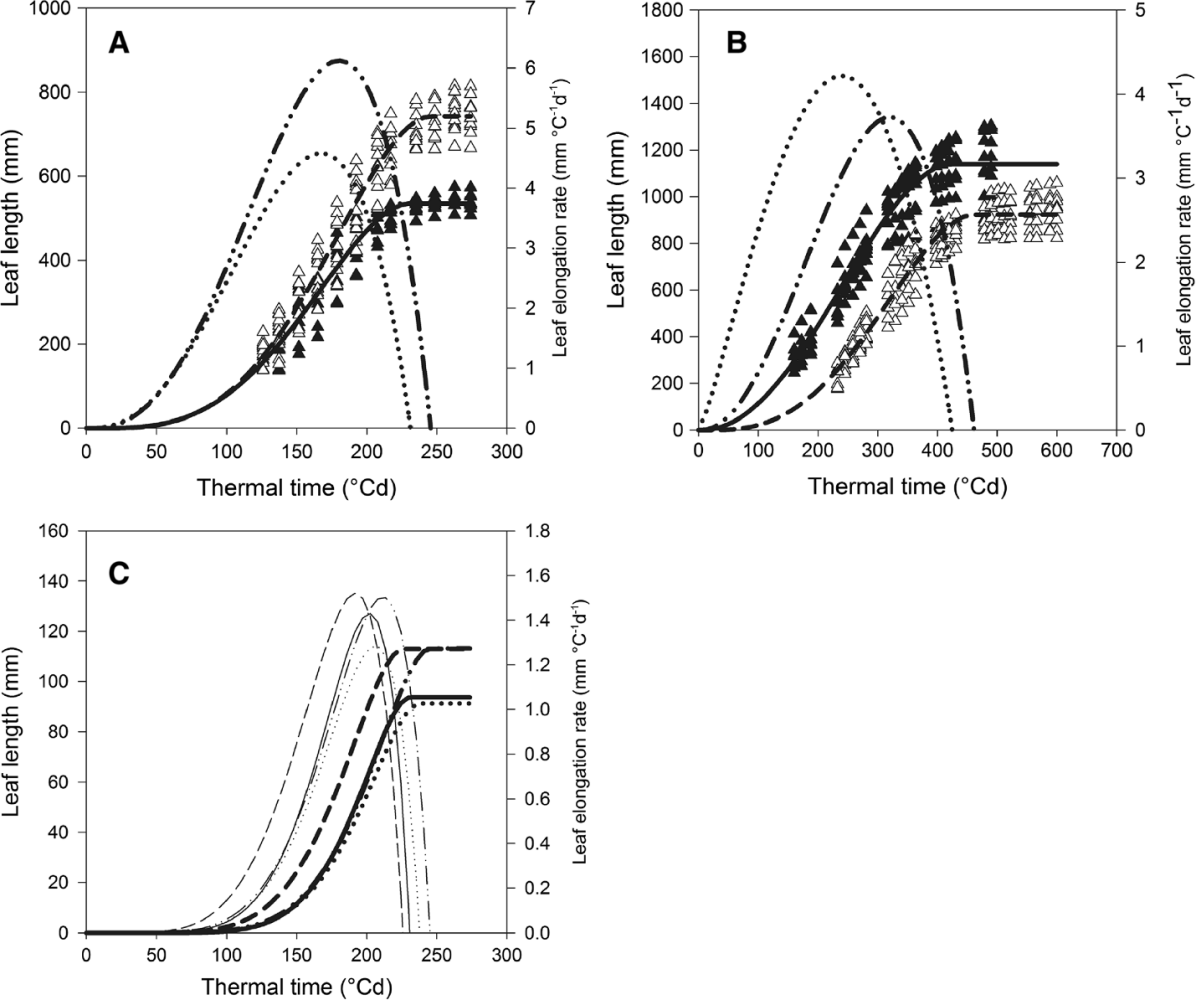
**Analysis of the leaf elongation datasets of maize,** ***Miscanthus*** **and** ***Brachypodium*** **using LEAF-E. (A)** Leaf length measurements of transgenic (white triangle) and non-transgenic (black triangle) plants of a segregating population produced by backcrossing a transgenic plant overexpressing the *GA20ox1 gene* to the wild-type line B104 maize, including leaf length (s-shaped) and LER (bell-shaped) function plots for both groups using the mean values of the function parameters. **(B)** Leaf length measurements of *Miscanthus sinensis* ‘Goliath’ (black triangle) and *M. x giganteus* (white triangle) plants, including leaf length and LER function plots for both groups using the mean values of the function parameters. **(C)** Leaf length and LER function plots for *Brachypodium distachyon* inbred lines Bd3-1 (full line), Bd21 (dotted), Bd21-3 (dashed) and Bd2-3 (dash dotted) using the mean values of the function parameters. For the sake of clarity, the individual leaf length measurements are not shown in this case.

Overexpression of *GA20ox1* in maize resulted into significantly longer leaves (*Lm*) and higher maximum leaf elongation rates (*LERmax*) (39% and 34% respectively, p < 0.001). This corresponded very well with the 38% increase in LER reported earlier by Nelissen and coworkers [28]. However, in addition to the calculation of *Lm* and *LERmax*, LEAF-E also facilitates analysis of parameters that describe the timing of leaf growth. For example, leaves of transgenic plants took slightly, but significantly more time to reach their full length (*te*) than those of non-transgenic plants (Figure 3A and Table 1). As a consequence, also *t50%*, *t90%* and *te*, as well as *tm*, occurred significantly later in the transgenics. This means that leaves of transgenic plants took slightly more time to reach 50%, 90% and 100% of their final leaf length.

Using LEAF-E to reanalyze dataset 1a, previous findings were confirmed but this analysis allowed a more detailed study of the timing of leaf growth, thereby facilitating the detection of dissimilarities such as a shift in attaining *LERmax* in *GA20ox1* overexpressing plants, that could not be quantified previously.

### Variation in leaf growth behavior in two *Miscanthus*species

We investigated the leaf growth characteristics of two genotypes of *Miscanthus* belonging to different species with high potential as bio-energy crops, but with contrasting phenotypic characteristics. *M. sinensis ‘Goliath’* is characterized by high shoot densities, whereas *M. x giganteus* produces less, but thicker and taller shoots [29, 30].

When comparing leaf growth characteristics of *M. sinensis ‘Goliath’* and *M. x giganteus*, we found that *M. sinensis ‘Goliath’* had significantly longer leaves than *M. x giganteus* (Table 2, Figure 3B). The leaves of *M. sinensis ‘Goliath’* on average attained a length of 1140 ± 46 mm, whereas leaves of *M. x giganteus* on average became 923 ± 26 mm in length (Table 2). The longer leaves of *M. sinensis ‘Goliath’* plants cannot be explained by significant changes in *LERmax* nor by an extended elongation period (no significant differences for LED values). However, our analysis revealed that leaves of *M. sinensis ‘Goliath’* plants display a very strong initial growth compared to *M. x giganteus*. Parameters *t20%*, *t50%* and *tm* are all attained sooner (Table 2). Leaves of *M. sinensis ‘Goliath’* reached 50% of their final leaf length at 230 ± 8°Cd, or 64°Cd sooner than *M. x giganteus* plants, at which point the leaves of the last were just emerging from the whorl.

**Table 2:** **Comparison of leaf elongation in two** ***Miscanthus*** **genotypes from different species**

Growth parameter			***M. Sinensis***‘Goliath’ (mean ± SE)	***M.***x ***giganteus***(mean ± SE)	Difference in mean ^(+)^	
	Lm	(mm)	1140 ± 46	923 ± 26	-217	**
	LERmax	(mm°C^-1^d^-1^)	4.2 ± 0.2	3.8 ± 0.2	-0.4	NS
Thermal time points	t100	(°Cd)	95 ± 4	167 ± 4	71	***
	t20%	(°Cd)	141 ± 7	207 ± 5	65	***
	t50%	(°Cd)	230 ± 8	294 ± 8	64	***
	tm	(°Cd)	240 ± 10	320 ± 8	80	***
	t90%	(°Cd)	350 ± 10	400 ± 13.8	50	NS
	te	(°Cd)	425 ± 10	461 ± 18	37	NS
Leaf elongation duration	LED(100-e)	(°Cd)	329 ± 8	295 ± 18	-35	NS
	LED(20%-90%)	(°Cd)	209 ± 5	194 ± 11	-15	NS
	LED(20%-e)	(°Cd)	283 ± 7	255 ± 16	-29	NS

The results are based on the analysis of nine *M. sinensis* ‘Goliath’ and eight *M.* x *giganteus* plants. Lm: final leaf length; LERmax: maximal leaf elongation rate; t20%, t50%, t90%, te: time points at which the leaf reaches 20**%**, 50**%**, 90**%** and 100**%** of the final leaf length, respectively; tm: time point at which the leaf reaches LERmax; LEDs: leaf elongation durations between above stated thermal time points.

+Statistical significance based on student t-test of *M. sinensis* ‘Goliath’ (n = 9) vs *M.* x *giganteus* (n = 8), **p < 0.01, ***p < 0.001, NS non-significant. Applied base temperature for thermal time calculation = 8°C, Mean of overall R^2^ values = 0.9932 (0.9695-0.9989).

The analysis with LEAF-E shows that the leaf growth pattern in these two *Miscanthus spp.* is significantly different and that the differences in total leaf length can be attributed to contrasting early leaf growth.

### Variation in leaf growth behavior in different *Brachypodium distachyon*inbred lines

Fifty *Brachypodium distachyon* inbred lines are currently being used for a study of natural diversity, which is led by ‘The International Brachypodium Initiative’ [23]. We analyzed the leaf growth behavior of four diploid *Brachypodium* inbred lines that are part of that study: Bd21, Bd21-3, Bd2-3 and Bd3-1 (Figure 3C).

Leaf growth analysis of these four genotypes with LEAF-E revealed distinct leaf growth characteristics. Based upon final leaf length, two groups can be distinguished. Bd21 and Bd3-1 have short leaves and Bd21-3 and Bd2-3 have long leaves (Figure 3C, Table 3). The length of the leaves is determined by both LER and LED. For Bd21, a low LER is probably the underlying factor of the shorter leaves (Table 3). This is in contrast to Bd3-1 which, like Bd21, has short leaves but a *LERmax* that is similar to those of the genotypes with longer leaves (Table 3). Parameters for LED are the smallest for Bd3-1. Thus, the leaf of Bd3-1 is short, most likely due to a short growing period. Bd21-3 and Bd2-3 both have long leaves, a high *LERmax* and similar LED. Despite these similarities, based upon our analysis, we can conclude that the 3^rd^ leaf of Bd21-3 plants starts and finishes its growth significantly earlier in thermal time than that of Bd2-3 (Table 3). These results suggest that different mechanisms might drive leaf growth in different accessions. In *Arabidopsis* it was found that at least five different mechanisms contribute to final leaf size [31].

**Table 3:** **Comparison of leaf elongation in four** ***Brachypodium*** **inbred lines**

Growth parameter			Bd3-1 (mean ± SE) ^(+)^	Bd21 (mean ± SE) ^(+)^	Bd21-3 (mean ± SE) ^(+)^	Bd2-3 (mean ± SE) ^(+)^
	Lm	(mm)	94^a^ ± 3	91^a^ ± 1	113^b^ ± 3	113^b^ ± 2
	LERmax	(mm°C^-1^d^-1^)	1.44^ab^ ± 0.05	1.29^a^ ± 0.03	1.52^b^ ± 0.05	1.51^b^ ± 0.03
Thermal time points	t20%	(°Cd)	162 ^a^ ± 3	163^a^ ± 2	148^b^ ± 3	166^a^ ± 2
	t20	(°Cd)	164^a^ ± 3	166^a^ ± 2	145^b^ ± 3	163^a^ ± 2
	t50%	(°Cd)	189^a^ ± 2	193^a^ ± 2	178^b^ ± 3	197^a^ ± 2
	tm	(°Cd)	202^a^ ± 2	206^a^ ± 2	192^b^ ± 3	211^a^ ± 2
	t90%	(°Cd)	217^ab^ ± 1	222^bc^ ± 2	210^a^ ± 4	229^c^ ± 2
	te	(°Cd)	230.8^ab^ ± 0.8	238^bc^ ± 2	226^a^ ± 4	245^c^ ± 2
Leaf elongation durations	LED(20-e)	(°Cd)	67^a^ ± 3	72^a^ ± 1	81^b^ ± 2	82^b^ ± 2
	LED(20%-90%)	(°Cd)	69^a^ ± 2	75^ab^ ± 1	78^b^ ± 1	79^b^ ± 1
	LED(20%-e)	(°Cd)	55^a^ ± 2	59^ab^ ± 1	62^b^ ± 1	63^b^ ± 1

Bd3-1 (n = 7), Bd21 (n = 10) plants, Bd21-3 (n = 7) plants, Bd2-3 (n = 10). Lm: final leaf length; LERmax: maximal leaf elongation rate; t20%, t50%, t90%, te: time points at which the leaf reaches 20%, 50%, 90% and 100% of the final leaf length, respectively; tm: time point at which the leaf reaches LERmax; LEDs: leaf elongation durations between above stated thermal time points.

+Statistical significance indicated with distinct letters based on ANOVA and Scheffé Post hoc test (p < 0.05) between lines Bd3-1 (n = 7), Bd21 (n = 10) plants, Bd21-3 (n = 7) plants, Bd2-3 (n = 10), applied base temperature for thermal time calculation = 11°C, Mean of overall R^2^ values = 0.9993 (0.9871-0.9993).

### Fitting of cell length measurements along the leaf axis of maize overexpressing *GA20ox1*using the beta sigmoid function

**Figure 4:**
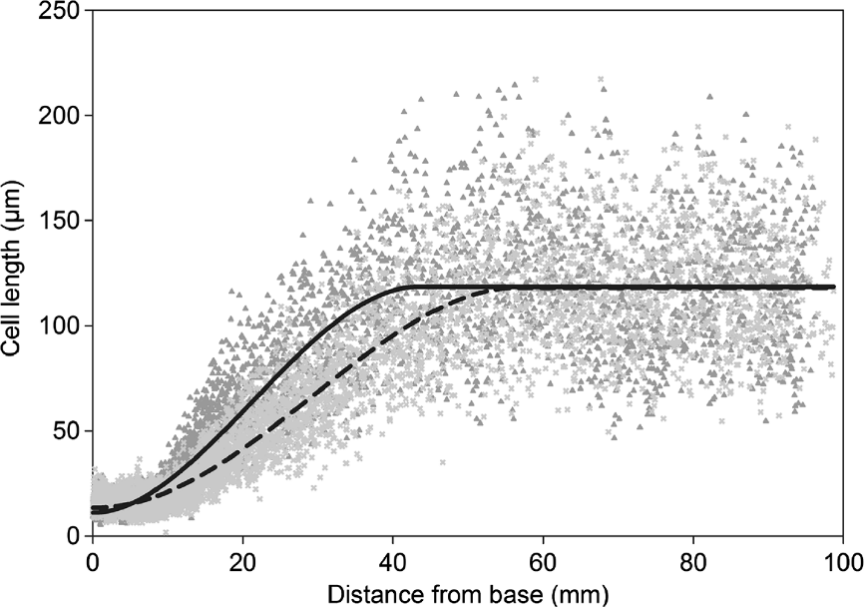
**Effect of** ***GA20ox1*** **overexpression on the cell length profile of the 4** ^**th**^ **leaf in maize.** The cell length profile along the axis of the 4^th^ leaf is shown for three non-transgenic (triangle, dark gray) and three transgenic (x, light gray) plants. The S-shaped curves are function plots using the mean values of the fitted function parameters for non-transgenic (full) and transgenic (dashed) profiles.

The cell length profile along the longitudinal axis of an actively growing grass leaf also displays a sigmoid pattern [8]. This sigmoidal profile is determined by the spatial distribution of cells in different stages of differentiation along the leaf axis: a number of dividing cells of small size at the leaf base, a stretch of cells that undergo elongation and thus increase in length when being pushed towards the leaf tip, and finally the tip of the leaf that is made up of cells that have reached their final length. We applied LEAF-E, adapted to use the extended version of the beta sigmoid function (Equation 3), to fit cell length measurements of dataset 1b. We found that fitting was successful and resulted in overall R²-values ranging from 0.8420 up to 0.8749 for cell length measurements of the *GA20ox1* overexpressing and control plants. Knowing that cell lengths can vary considerably, even for adjacent cells of the same cell file in one leaf (Figure 4), these R²-values are noticeably high. However, a goodness of fit assessment on cell length measurements is, to our knowledge, not reported in literature, making any comparison difficult. We explored the suitability of three other commonly used sigmoidal functions, namely the Weibull, the Logistic and the Gompertz functions for fitting cell length data of the control plants. The fits of cell length data resulted in R²-values of 0.8504 ± 0.0054 for Weibull, 0.8485 ± 0.0055 for logistics, 0.8499 ± 0.0053 for beta sigmoid and 0.8495 ± 0.0054 for Gompertz. The goodness of fit was also evaluated by linear regression of the estimated to the measured values (Additional file 6) and again found highly similar R²-values. For the beta sigmoid function, we investigated the deviation of the data points from the fitted curve and could detect a slight underestimation of cell lengths in the very first 3 mm from the leaf base (Additional file 3D). This is because the cells in the first few millimeter of the division zone are slightly longer than those in the middle of the division zone; this hyperbolic profile at the leaf basis cannot be fitted with the functions that we used here, since they assume an increment in cell lengths. Additionally, the value of the residuals increased with the distance from the leaf base (Additional files 3D and 6). For this dataset, the use of the beta sigmoid function in the form of Equation 3 is preferred over the other investigated functions because the function parameters themselves provide relevant biological information. However, the exploration of mathematical functions that are more appropriate for fitting cell lengths along the longitudinal axis of grass leaves should be the topic of future research.

The cell length profile along the leaf axis in maize plants overexpressing *GA20ox1* (dataset 1b) was analyzed by Nelissen and coworkers [28] using a polynomial fit approach similar to Rymen and coworkers [3]. Using LEAF-E, we determined that the size of the division zone, defined as the stretch of cells near the leaf base by which function fitted cell lengths do not exceed 40 μm (obtained by DAPI-staining of cells along the proximal-distal axis), is on average 33% longer (p < 0.01) in *GA20ox1* overexpressing leaves as compared to non-transgenic leaves (Figure 4, Table 4). We estimated that the elongation zone, defined as the distance between the end of the division zone and the function parameter *Pe* (position in the leaf where cells reach their maximal length, equivalent to *te* for leaf elongation), is 29% longer (p < 0.01) in *GA20ox1* overexpressing leaves. Moreover, we can state that the position where maximal cell elongation occurs (*Pm*, equivalent to *tm* for leaf elongation) is situated 8 mm (or 38%) further away from the leaf base in *GA20ox1* overexpressing leaves as compared to non-transgenic plants and that the mature cell length *Lm* (mm) did not differ (p = 0.92). These findings are in accordance with results obtained earlier by Nelissen and coworkers [28]. The analysis using LEAF-E thus allowed a more detailed study of the cell length profile, which facilitated the detection of a longer elongation zone in *GA20ox1* overexpressing plants and a shift in the position of maximal cell elongation, that could not be quantified previously. Nevertheless, it might still be necessary to validate the estimation of the size of the division zone using DAPI staining or a similar method, as was performed here. We believe that function fitting, using LEAF-E, can be very useful and might even be a necessary step for the analysis of cell length profiles in the grass leaf in future experiments.

**Table 4:** **Effect of GA20ox1 overexpression on the maize cell length profile**

Parameter			***AtGA20ox1***OE (mean ± SE)	Control (mean ± SE)	Difference in mean ^(+)^	
Cell length	Lb	(μm)	13.2 ± 1.7	10.9 ± 0.3	22%	NS
	Lm	(μm)	118.1 ± 0.6	118.5 ± 3.8	0%	NS
Position along the leaf axis	Pm	(mm)	29.5 ± 2.1	21.3 ± 0.8	38%	**
	Pe	(mm)	56.6 ± 2.5	43.4 ± 0.3	-30%	**
Zones along the leaf axis	division zone (cell < =40 μm)	(mm)	19.5 ± 0.5	14.6 ± 0.3	33%	**
	elongation zone (cell > 40 μm - Pe)	(mm)	37.1 ± 1.0	28.7 ± 0.9	-29%	**

A segregating population produced by backcrossing (BC) a transgenic plant overexpressing the *Arabidopsis thaliana GIBBERELLIC ACID 20 OXIDASE1 (GA20ox1)* gene to the wild-type line B104 was analyzed for the cell lengths profile along the leaf axis. The results are based on the analysis of three transgenic and three non-transgenic BC1 plants. Lb: initial cell length Lm: final cell length; Pm: position along the leaf axis with maximal cell elongation rate; Pe: position along the leaf axis where cells reach their final length.

+Statistical significance based on student t-test of non-transgenic plants (n = 3) vs *GA20ox1* overexpression (n = 3), **p < 0.01, NS non-significant.

## Conclusions

Here, we provide a tool that we named LEAF-E for fast and straightforward analysis of grass leaf growth data using nonlinear regression in a simple Microsoft Excel format. We automated the fitting procedure using Excel 2010 and the Solver function (32 bit). The results of fitting the beta sigmoid function to the leaf length measurements are stored in tabular form and can easily be analyzed in standard statistical software programs in search of differences due to the applied treatments or to explore inter-genotypic or inter-population differences.

We applied LEAF-E to three datasets containing leaf length measurements of maize, *Miscanthus* and *Brachypodium*. In *Miscanthus* and *Brachypodium*, we have shown that LEAF-E is an appropriate tool for data analysis and that the analyzed species and genotypes display distinct leaf growth characteristics. In maize, the changes in both leaf elongation and cell length profile along the leaf axis as a result of enhanced GA levels, previously demonstrated by Nelissen and coworkers [28], were confirmed. In addition, we demonstrated that using LEAF-E, the timing of leaf growth can be studied, thereby facilitating the detection of dissimilarities in the timing of leaf growth that could not be quantified using other approaches. Furthermore, analysis with LEAF-E allows for a robust calculation of *LERmax* and LED, which leads to reliable detection of significant changes. Therefore, we propose LEAF-E as an excellent tool for comparing leaf growth behavior in different genotypes or to analyze the response of specific genotypes to a treatment.

## Methods

### Datasets used for validation

#### Dataset 1a

The dataset on leaf elongation of the maize B104 inbred line overexpressing the *Arabidopsis thaliana GIBBERELLIC ACID 20 OXIDASE1* (*GA20ox1*) gene, previously described by Nelissen and coworkers [28] was reanalyzed here. A segregating population produced by backcrossing the overexpression line (hemizygous for the transgenic event) to the wild-type B104 inbred line and consisting of 9 non-transgenic and 11 transgenic plants was used.

To determine leaf elongation rates, the length of the 4^th^ leaf of transgenic and non-transgenic plants was measured daily until complete development, as previously described [28]. The plants were grown in a growth chamber at 24°C. Here we used a base temperature of 10°C for thermal time (Growing degree days, GDD) calculations. For further details about growth conditions see [28].

#### Dataset 1b

The same segregating population, used to generate dataset 1a, was previously used by Nelissen and coworkers [28] for the analysis of cell lengths along the leaf axis, based upon methods previously described [32]. In short, the 4^th^ leaf was harvested two days after appearance from the pseudo-stem (stem-like structure composed of concentric rolled or folded blades and sheaths that surround the growing point). At this time point, the ligule is only a few mm away from the base of the plant. The length of cell files adjacent to stomatal rows along the proximal-distal axis was measured using a DIC microscope (AxioImager, Zeiss, USA), and image analysis software (AxioVision, Zeiss, USA). The size of the division zone was determined as the distance between the base and the most distally observed mitotic figure in DAPI-stained leaves along the proximal-distal axis, with a fluorescence microscope (AxioImager, Zeiss, USA). Here we reanalyzed the cell length measurements.

#### Dataset 2

Eight *Miscanthus* x *giganteus* and nine *M. sinensis* ‘Goliath’ plants were grown at 20°C (average temperature over the measuring period was 19.1°C) in a greenhouse, in Melle, Belgium, in September 2012 with no supplementary light. Plants were grown from rhizome cuttings in 2-l pots and were hand-watered and not fertilized during the experiment. The rhizomes were excavated during the winter of 2011 and stored in a cold room at 3°C until the start of the experiment. The length of the 4^th^ leaf was measured five times a week (from leaf tip to soil level). Based on our observations at this developmental stage, the contribution of internode elongation to the height measurements as leaf lengths can be neglected and transition to flowering has not yet occurred. The measurements were spread over a time period of approximately four weeks. The calculation of thermal time was based on the average air temperature in the greenhouse taking into account a base temperature of 8°C, based on Farrell and coworkers [33].

#### Dataset 3

The *Brachypodium distachyon* inbred lines Bd21, Bd2-3 and Bd3-1 were provided by David F. Garvin from the USDA-ARS (Minnesota, US), and line Bd21-3 was provided by Richard Sibout from INRA-IJPB (Versailles, France). Plants were grown in rootrainers (Haxnicks®, UK) in biological replicates (n = 10, 10, 7, 9 respectively) in a greenhouse at an average temperature of 21°C in Melle, Belgium, August 2012 with no supplementary light. To calculate the thermal time, a base temperature of 10°C was used. Fertilizer was added with the water supply: conductivity Ec = 1mS/cm; water soluble fertilizer Poly-feed (Haifa, Belgium) (N, P_2_O_5_, K_2_0; 20:5:20 + 3 MgO). Measurements were taken from the tip of the 3^rd^ leaf to its basal level on a daily basis, for a period of 10 days. Based on our observations at this developmental stage, the contribution of internode elongation to the height measurements as leaf lengths can be neglected and transition to flowering has not yet occurred.

### A mathematical function for fitting leaf length measurements

For the estimation of leaf growth parameters we used the beta sigmoid function for determinate growth, inspired by the Euler integral, in the form of Equation 1. This function was used previously by Auzanneau and coworkers [7] and Verdenal and coworkers [19] to model leaf growth after cutting in *Lolium perenne*. In short, the leaf length *L* (mm) at a given moment in development *t* (°Cd) is determined by final leaf length *Lm* (mm) and three particular points in leaf development, expressed as units of thermal time or growing degree days (°Cd). Thermal time is a summation of cumulative differences between daily mean temperature and a specified base temperature, below which the plant does not grow or grows very slowly [22]. These thermal time points are the moment at which leaf growth starts *t0* (°Cd), the moment of maximal leaf growth rate *tm* (°Cd) and the moment at which leaf growth ceases *te* (°Cd). Estimations of *t0* often result in negative values that are biologically not relevant [7]. Therefore, in the experiments in which seedlings were involved (maize and *Brachypodium*), we assumed that *t0* = 0 was at the moment of sowing. In the case of *Miscanthus*, *t0* = 0 was assumed to be at the moment of potting the rhizomes (no visible leaves at this stage).
1

Equation 1. Beta sigmoid function for fitting leaf length, modified from [7]. Function is applicable for *t0* ≤ *t* ≤ *te* and *t0* ≤ *tm* < *te*. For *t* > *te*, Equation 1 is reduced to *L* = *Lm*.

The leaf elongation rate (LER) at any given moment in leaf development *t* (°Cd) can be calculated from the LER function (Equation 2), which is the first derivative of Equation 1. From this equation we determined the maximum leaf elongation rate or *LERmax* (mm/°Cd), as the LER at *tm*.
2

Equation 2. Leaf elongation rate function, modified from [7].

As Equation 1 is a continuous function, it allows calculating the leaf length *L* (mm) at any given moment in the leaf elongation period *t*, and vice versa (Figure 2). Therefore, in addition to the parameters *Lm*, *t0*, *tm* and *te*, we estimated a set of parameters that can be biologically relevant. For the maize dataset 1a we estimated the time point *t100*, the moment at which the leaf length is 100 mm. The *t100* time point, which is early in development, was chosen since it is close to the moment at which the leaf emerges from the pseudo-stem in maize non-transgenic B104 plants. Furthermore, we estimated *t20%*, *t50%* and *t90%* (°Cd), which are the moments at which the leaf reaches 20%, 50% and 90% of its final length, respectively. For *Brachypodium* (dataset 2), we replaced the *t100* parameter by *t20* (°Cd), the moment at which the leaf reaches 20 mm in length, to accommodate the smaller size of the *Brachypodium* leaf. These extra parameters allow the comparison of responses to different treatments or detect inter-genotypic differences in leaf growth development.

Expressing leaf growth as durations of thermal time, the leaf elongation duration or LED, allows describing leaf growth in a fluctuating environment [15].

On that account, various LEDs were explored. For example, *LED(20%-90%)* defines the leaf growth duration between reaching 20% and 90% of its final length. Parameters *LED(100-e)* and *LED(20%-e)* were defined similarly.

### A mathematical function for fitting cell length measurements

Often, analysis of leaf growth involves cell length measurements along the leaf axis, providing insight in the sizes of dividing, elongating and mature cells, and of the leaf zones encompassing these three cell types [3, 8, 9, 15, 24, 25]. Therefore, we assessed the fitting of cell length measurements along the leaf axis in maize with the beta sigmoid function. However, since in this case cells in the division zone have an initial length before proceeding to the elongation phase and eventually toward mature cells, a different equation had to be used as Equations 1 and 2 assume zero values at the beginning of growth. Yin and coworkers [18] describe an extended version of the beta sigmoid function that allows taking into account the initial length of the cell, *Lb* (mm) (Equation 3). In this case the data points do not represent a time series, but a positional-series of cell lengths along the leaf axis, taking the leaf base as position zero. For ease of interpretation, the symbols of the variable *t* and parameters *tm* and *te* from the original equation have been converted into the positions *p* (mm) along the leaf axis, starting from the base towards the tip. The position at which maximal cell elongation occurs is denoted as *Pm* (mm), and the position at which the cells cease to elongate is denoted as *Pe* (mm) (see Equation 3).
3

Equation 3. Extended version of the beta sigmoid function modified from [18] to fit cell length measurements along the leaf axis.

### LEAF-E: function fitting using a Microsoft excel spreadsheet and the SOLVER function

The fitting of leaf growth data using the beta sigmoid function was performed using Excel 2010 and the Solver function (32 bit) according to Brown [34]. The automation of the procedure, as described below, in the form of a macro is innovative. Each row in the datasheet contains the data of one individual leaf (ordered in a time series), the starting values of the parameters of the model, and the formulae to extract the necessary statistical components for the calculation of the least square estimates following Neter and coworkers [35]. First, the macro checks for non-empty rows. When a non-empty row is found, the model is fitted to the data by minimizing the sum of squares of the errors iteratively, and changing the starting values of the parameters at each step. Per row (=leaf), all values described in the previous sections are calculated and stored in a tabular form for further statistical analysis. In addition, a graph showing the original data points, the fitted growth curve and the function parameters is generated automatically. This enables the evaluation of the correlation coefficient and visual interpretation of the goodness of fit. It also provides an easy way to check for miss fits or errors in the data. Miss fitting can occur when the Solver function fails to minimize the sum of squares of the errors using a particular set of starting values. Accordingly, the procedure can be repeated using more appropriate starting values. This way of working guarantees a fast analysis, robust estimation of growth parameters and provides a table in standardized format containing the resulting parameters and derived parameters per leaf. The output data can easily be analyzed in search of differential responses using standard statistical software, depending on the experimental setup. We gave this Excel tool the name LEAF-E. LEAF-E is included as additional file to this article (Additional files 4 and 5, with a user manual on the first sheet). Additional file 4 includes test data of *M. sinensis* ‘Goliath’ leaf growth and default starting values prior running the macro and Additional file 5 includes the results of the fitting procedure and graphs after running the automated fitting procedure.

### Statistical analysis

The statistical analysis of the derived growth parameters comprised a student t-test on datasets containing only two genotypes (datasets 1a, 1b and 2) and an ANOVA followed by post hoc Scheffé tests for datasets containing more than two genotypes (dataset 3). All these analyses were carried out in the software package *STATISTICA* version 11 (Statsoft Inc., USA). For the analysis of the goodness of fit of cell length data of dataset 1b, the fitting of the beta sigmoid and three other commonly used growth functions (Weibull, Gompertz and Logistic) and linear regression of the predicted to the observed values was carried out in *SIGMAPLOT* version 12_5 (Systat Software Inc., USA).

## Electronic supplementary material

Additional file 1: **Function fitting of leaf length measurements of maize,** ***Miscanthus*** **and** ***Brachypodium*** **using LEAF-E on a plant-by-plant basis.** The PowerPoint presentation shows a plot for every single plant of every dataset, showing the individual leaf length measurements, the fit of the beta sigmoid function and its R²-value, the estimated LER curve and the calculated LER (calculated as leaf length increase between two consecutive measurements divided by the respective thermal time interval). Maize GA: transgenic plant overexpressing the *GA20ox1 gene*, Maize control: wild-type B104 line, Bd: *Brachypodium distachyon*, F(t): fitted curve plotted in thermal time, R² F(t): R² value of the fit, Fler(t): LER curve, plotted in thermal time, LER calc: calculated LER (see above). (PPTX 575 KB)

Additional file 2: **Scatter plots and linear regression for datasets of leaf length measurements.** Plots show estimated to measured leaf lengths (A, D, G), estimated LER to calculated LER (calculated as leaf length increase between two consecutive measurements divided by the respective thermal time interval) (B, E, H) and estimated *LERmax* to *LERmax* determined as maximal value of the profile of calculated LER (C, F, I), all on a plant-by-plant basis, for datasets of maize (dataset 1a: A, B, C), *Miscanthus* (dataset 2: D, E, F) and *Brachypodium* (dataset 3: G, H, I). (TIFF 213 KB)

Additional file 3: **Distribution of the residuals for the fitting of the beta sigmoid function to leaf length data of maize (A),** ***Miscanthus*** **spp. (B) and** ***Brachypodium*** **spp. (C) and cell length data of maize (D) using LEAF-E.** (JPEG 2 MB)

Additional file 4: **The Microsoft excel spreadsheet named LEAF-E with test data (** ***M. sinensis*** **‘Goliath leaf length measurements) and default starting values prior running the automated fitting procedure.** (XLSM 975 KB)

Additional file 5: **The Microsoft excel spreadsheet named LEAF-E with test data (** ***M. sinensis*** **‘Goliath leaf length measurements), graphs and results of the fitted growth parameters after running the automated fitting procedure.** The first sheet contains a user manual. (XLSM 1 MB)

Additional file 6: **Comparison of goodness of fit of the Beta sigmoid (A), Weibull (B), Gompertz (C) and Logistic (D) functions for the cell length profile of the 4** ^**th**^ **leaf in maize.** The plots show the linear regression of estimated versus measured cell lengths of all data points of dataset 1b. (JPEG 3 MB)
