# Supplementary material for: LEAF-E: a tool to analyze grass leaf growth using function fitting
Source: Plant Methods. 2014 Nov 6;10:37. doi: 10.1186/1746-4811-10-37 (PMC4246515; doi:10.1186/1746-4811-10-37)
Supplement: Supplementary file 1 — Additional file 1: Function fitting of leaf length measurements of maize, Miscanthus and Brachypodium using LEAF-E on a plant-by-plant basis. The PowerPoint presentation shows a plot for every single plant of every dataset, showing the individual leaf length measurements, the fit of the beta sigmoid function and its R²-value, the estimated LER curve and the calculated LER (calculated as leaf length increase between two consecutive measurements divided by the respective thermal time interval). Maize GA: transgenic plant overexpressing the GA20ox1 gene, Maize control: wild-type B104 line, Bd: Brachypodium distachyon, F(t): fitted curve plotted in thermal time, R² F(t): R² value of the fit, Fler(t): LER curve, plotted in thermal time, LER calc: calculated LER (see above). (PPTX 575 KB) [file 13007_2014_305_MOESM1_ESM.pptx]

## Slide 1
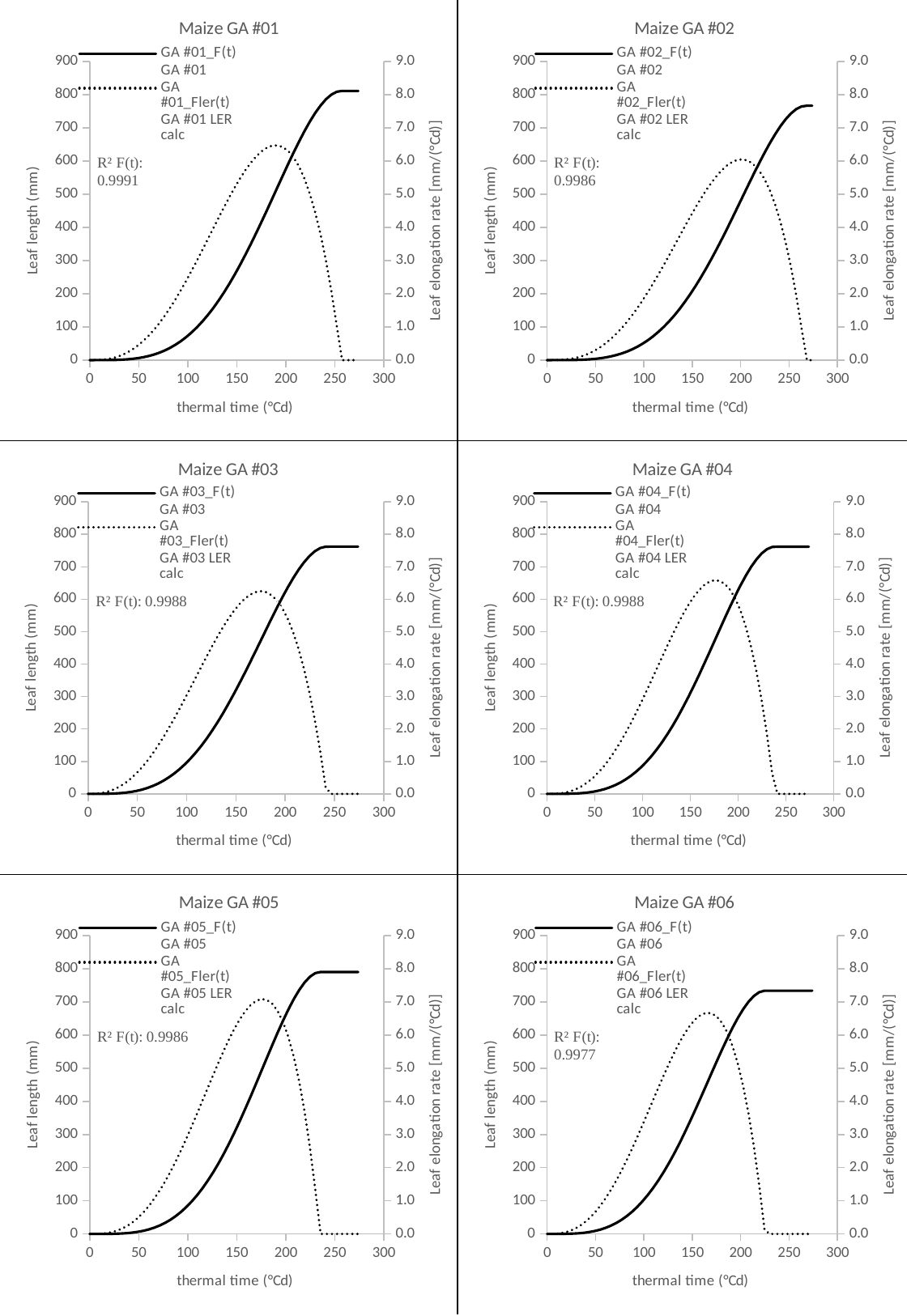

### Chart: Maize GA #01
| Category | GA #01_F(t) | GA #01 | GA #01_Fler(t) | GA #01 LER calc |
|---|---|---|---|---|
### Chart: Maize GA #02
| Category | GA #02_F(t) | GA #02 | GA #02_Fler(t) | GA #02 LER calc |
|---|---|---|---|---|
### Chart: Maize GA #03
| Category | GA #03_F(t) | GA #03 | GA #03_Fler(t) | GA #03 LER calc |
|---|---|---|---|---|
### Chart: Maize GA #04
| Category | GA #04_F(t) | GA #04 | GA #04_Fler(t) | GA #04 LER calc |
|---|---|---|---|---|
### Chart: Maize GA #05
| Category | GA #05_F(t) | GA #05 | GA #05_Fler(t) | GA #05 LER calc |
|---|---|---|---|---|
### Chart: Maize GA #06
| Category | GA #06_F(t) | GA #06 | GA #06_Fler(t) | GA #06 LER calc |
|---|---|---|---|---|

## Slide 2
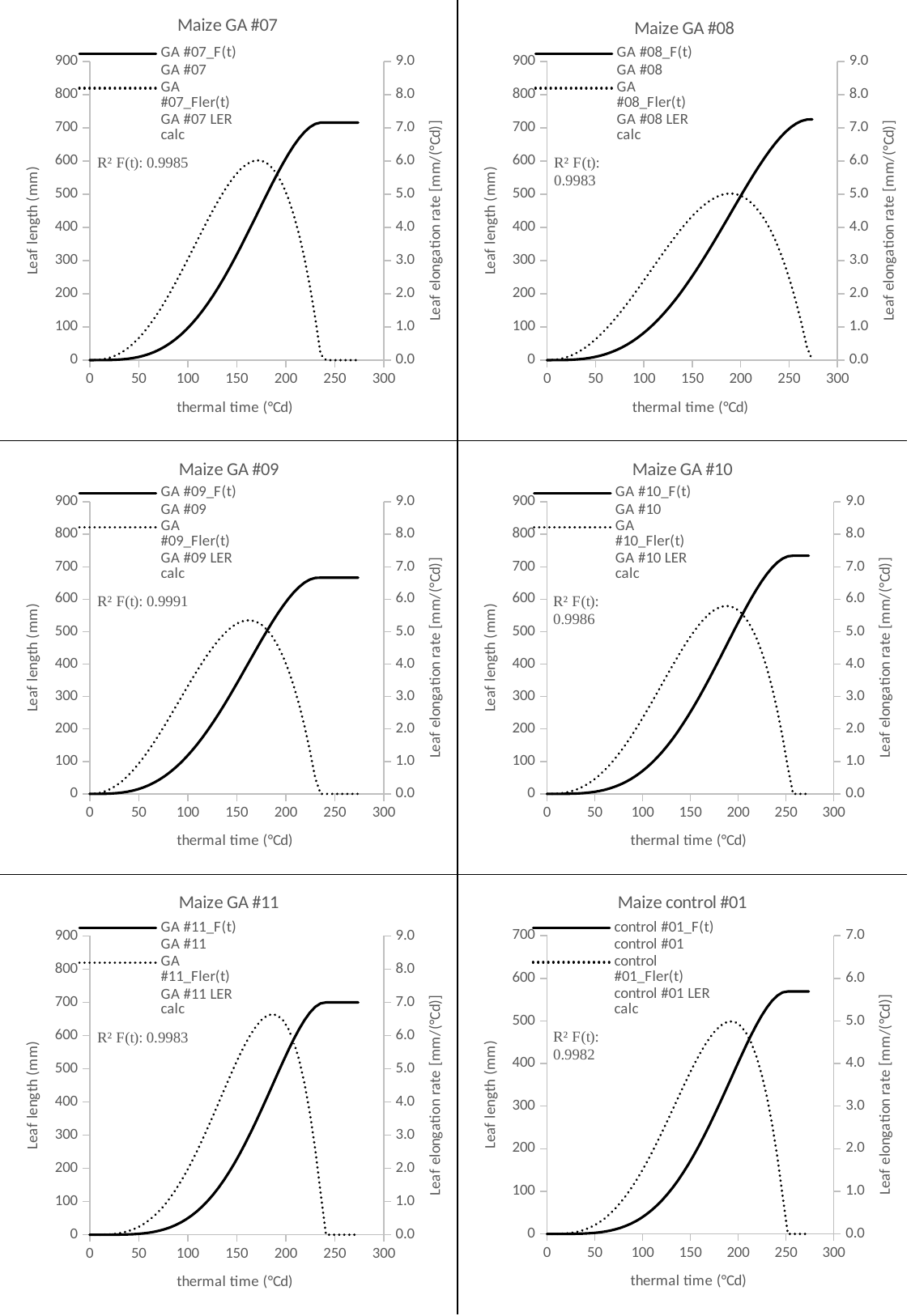

### Chart: Maize GA #07
| Category | GA #07_F(t) | GA #07 | GA #07_Fler(t) | GA #07 LER calc |
|---|---|---|---|---|
### Chart: Maize GA #08
| Category | GA #08_F(t) | GA #08 | GA #08_Fler(t) | GA #08 LER calc |
|---|---|---|---|---|
### Chart: Maize GA #09
| Category | GA #09_F(t) | GA #09 | GA #09_Fler(t) | GA #09 LER calc |
|---|---|---|---|---|
### Chart: Maize GA #10
| Category | GA #10_F(t) | GA #10 | GA #10_Fler(t) | GA #10 LER calc |
|---|---|---|---|---|
### Chart: Maize control #01
| Category | control #01_F(t) | control #01 | control #01_Fler(t) | control #01 LER calc |
|---|---|---|---|---|
### Chart: Maize GA #11
| Category | GA #11_F(t) | GA #11 | GA #11_Fler(t) | GA #11 LER calc |
|---|---|---|---|---|

## Slide 3
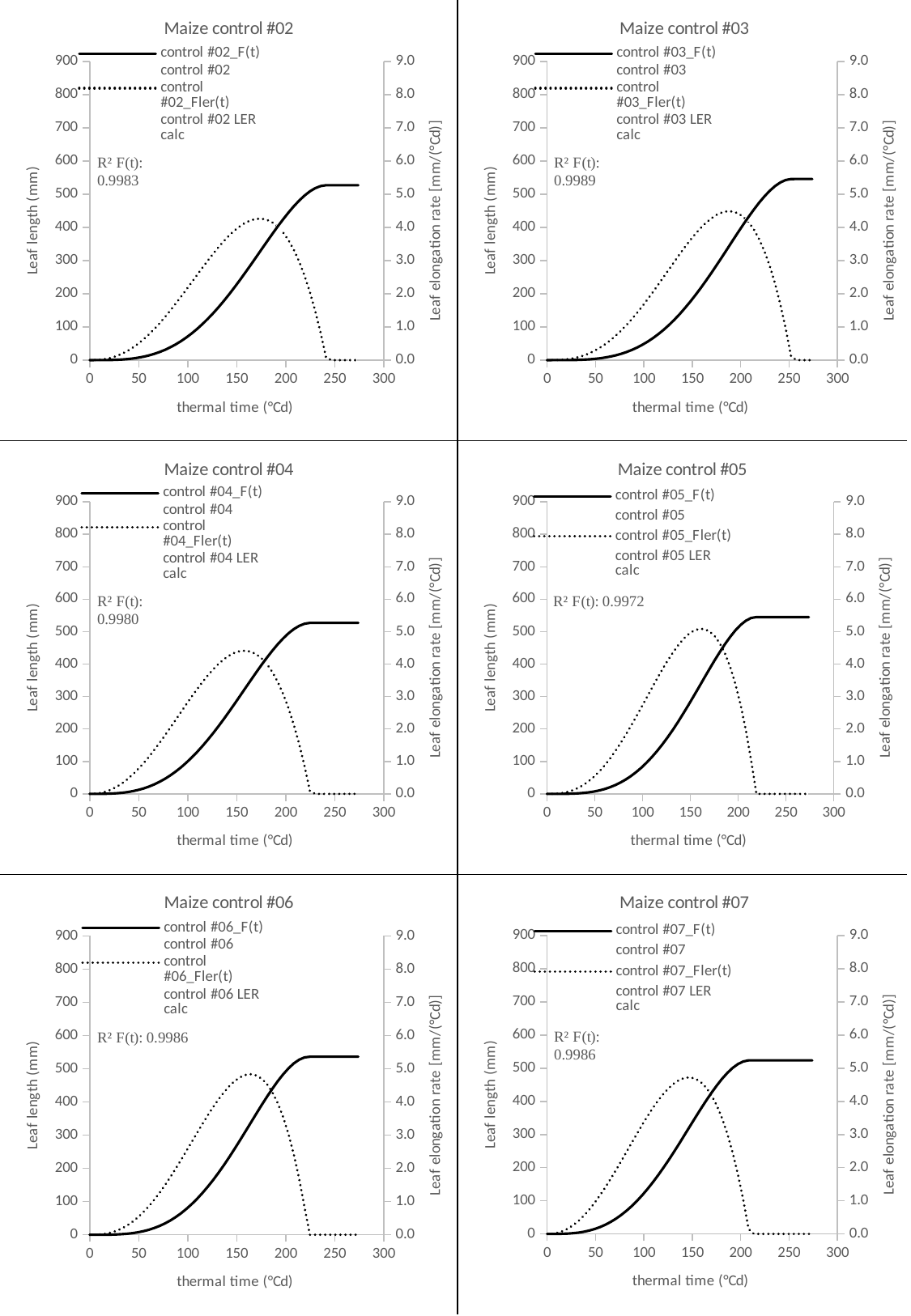

### Chart: Maize control #02
| Category | control #02_F(t) | control #02 | control #02_Fler(t) | control #02 LER calc |
|---|---|---|---|---|
### Chart: Maize control #03
| Category | control #03_F(t) | control #03 | control #03_Fler(t) | control #03 LER calc |
|---|---|---|---|---|
### Chart: Maize control #04
| Category | control #04_F(t) | control #04 | control #04_Fler(t) | control #04 LER calc |
|---|---|---|---|---|
### Chart: Maize control #05
| Category | control #05_F(t) | control #05 | control #05_Fler(t) | control #05 LER calc |
|---|---|---|---|---|
### Chart: Maize control #06
| Category | control #06_F(t) | control #06 | control #06_Fler(t) | control #06 LER calc |
|---|---|---|---|---|
### Chart: Maize control #07
| Category | control #07_F(t) | control #07 | control #07_Fler(t) | control #07 LER calc |
|---|---|---|---|---|

## Slide 4
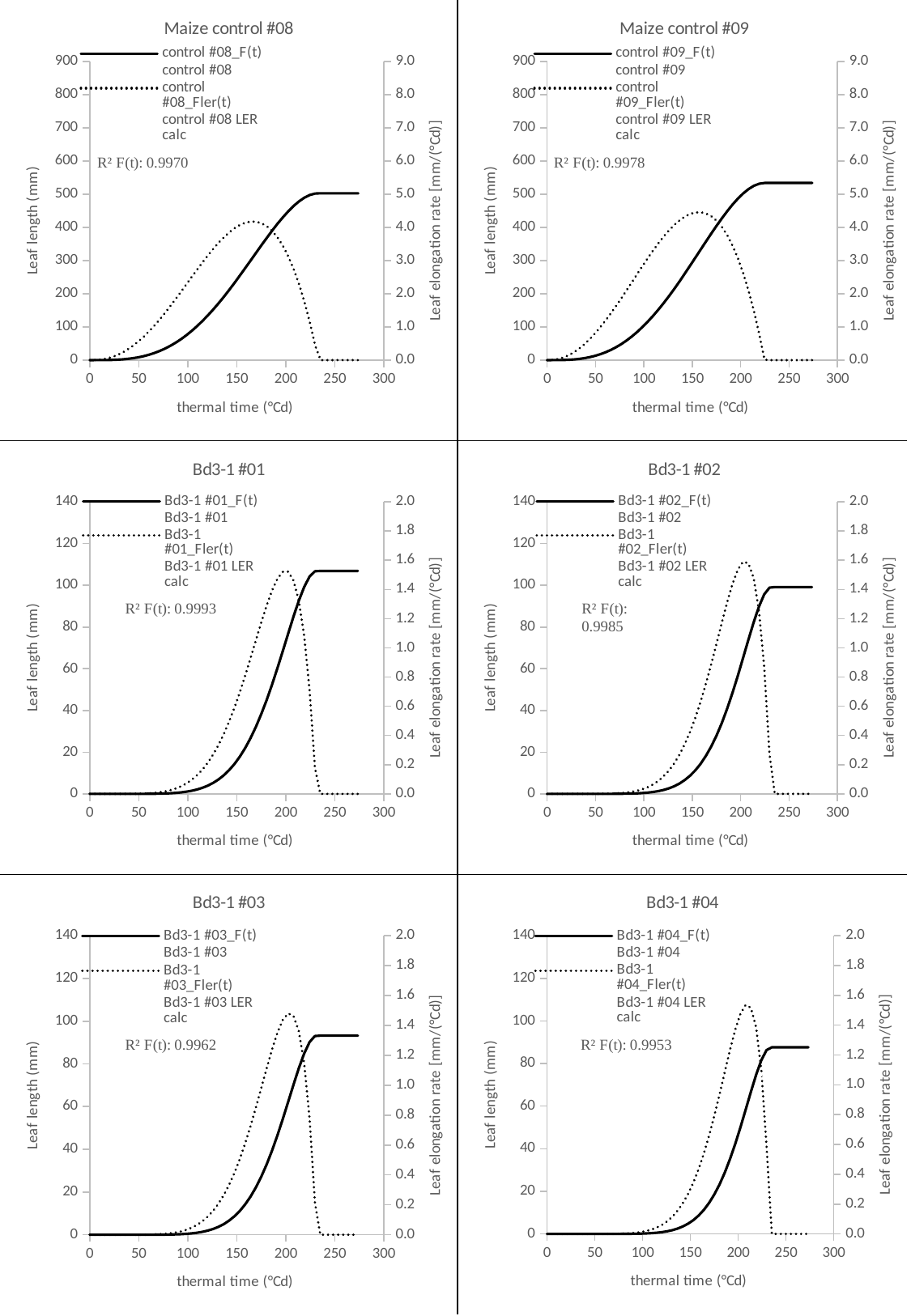

### Chart: Maize control #08
| Category | control #08_F(t) | control #08 | control #08_Fler(t) | control #08 LER calc |
|---|---|---|---|---|
### Chart: Maize control #09
| Category | control #09_F(t) | control #09 | control #09_Fler(t) | control #09 LER calc |
|---|---|---|---|---|
### Chart: Bd3-1 #01
| Category | Bd3-1 #01_F(t) | Bd3-1 #01 | Bd3-1 #01_Fler(t) | Bd3-1 #01 LER calc |
|---|---|---|---|---|
### Chart: Bd3-1 #02
| Category | Bd3-1 #02_F(t) | Bd3-1 #02 | Bd3-1 #02_Fler(t) | Bd3-1 #02 LER calc |
|---|---|---|---|---|
### Chart: Bd3-1 #03
| Category | Bd3-1 #03_F(t) | Bd3-1 #03 | Bd3-1 #03_Fler(t) | Bd3-1 #03 LER calc |
|---|---|---|---|---|
### Chart: Bd3-1 #04
| Category | Bd3-1 #04_F(t) | Bd3-1 #04 | Bd3-1 #04_Fler(t) | Bd3-1 #04 LER calc |
|---|---|---|---|---|

## Slide 5
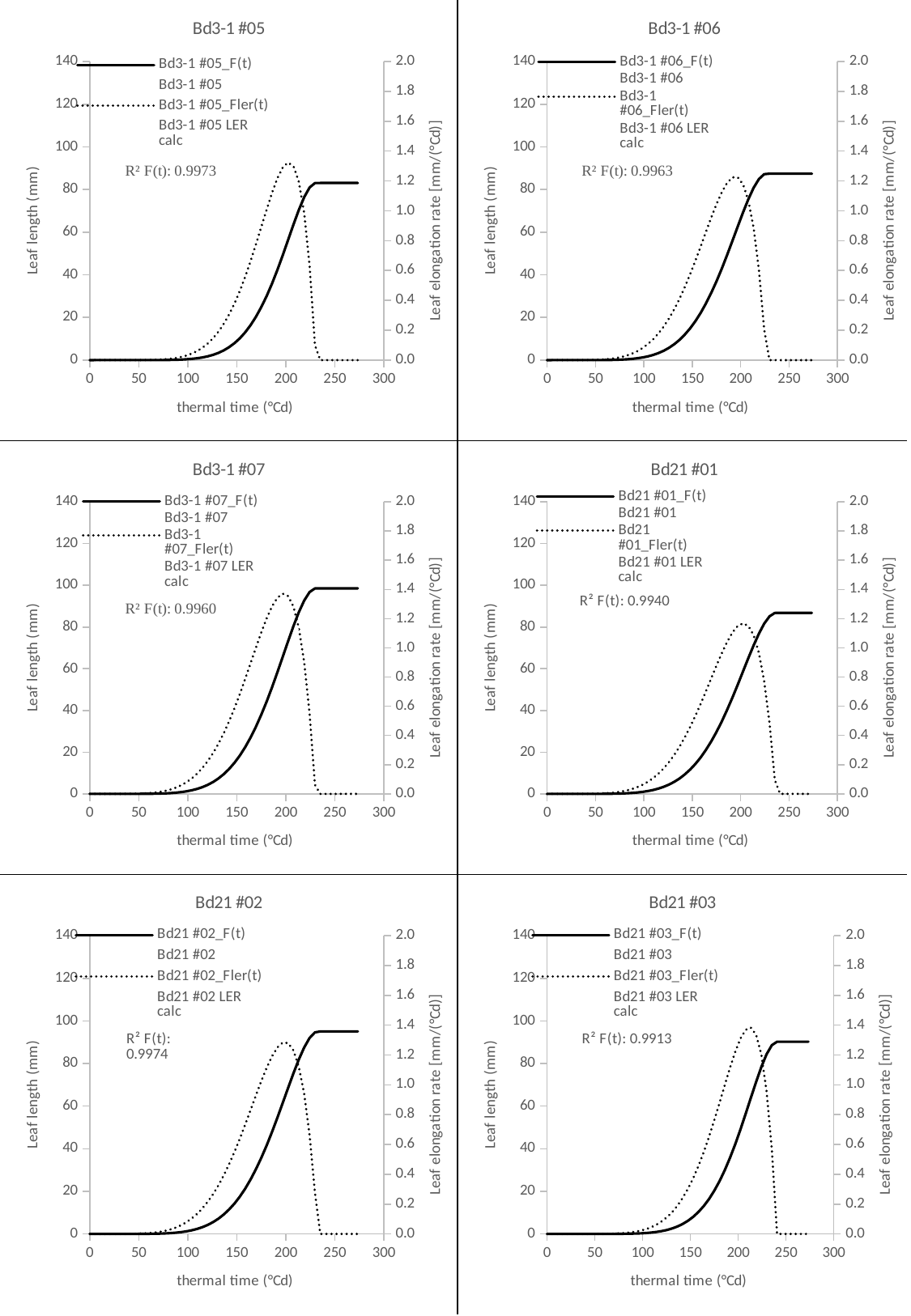

### Chart: Bd3-1 #05
| Category | Bd3-1 #05_F(t) | Bd3-1 #05 | Bd3-1 #05_Fler(t) | Bd3-1 #05 LER calc |
|---|---|---|---|---|
### Chart: Bd3-1 #06
| Category | Bd3-1 #06_F(t) | Bd3-1 #06 | Bd3-1 #06_Fler(t) | Bd3-1 #06 LER calc |
|---|---|---|---|---|
### Chart: Bd3-1 #07
| Category | Bd3-1 #07_F(t) | Bd3-1 #07 | Bd3-1 #07_Fler(t) | Bd3-1 #07 LER calc |
|---|---|---|---|---|
### Chart: Bd21 #01
| Category | Bd21 #01_F(t) | Bd21 #01 | Bd21 #01_Fler(t) | Bd21 #01 LER calc |
|---|---|---|---|---|
### Chart: Bd21 #02
| Category | Bd21 #02_F(t) | Bd21 #02 | Bd21 #02_Fler(t) | Bd21 #02 LER calc |
|---|---|---|---|---|
### Chart: Bd21 #03
| Category | Bd21 #03_F(t) | Bd21 #03 | Bd21 #03_Fler(t) | Bd21 #03 LER calc |
|---|---|---|---|---|

## Slide 6
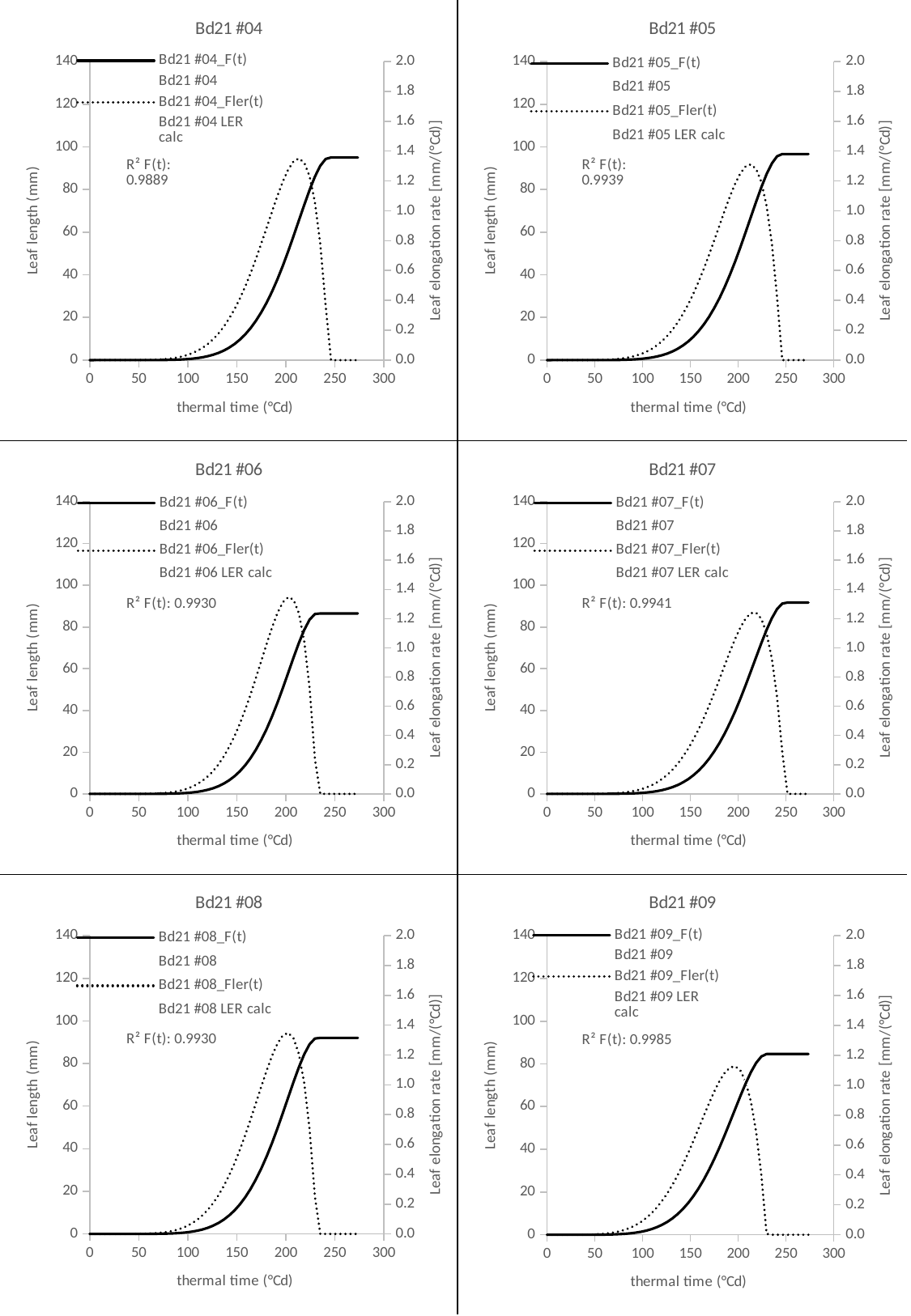

### Chart: Bd21 #04
| Category | Bd21 #04_F(t) | Bd21 #04 | Bd21 #04_Fler(t) | Bd21 #04 LER calc |
|---|---|---|---|---|
### Chart: Bd21 #05
| Category | Bd21 #05_F(t) | Bd21 #05 | Bd21 #05_Fler(t) | Bd21 #05 LER calc |
|---|---|---|---|---|
### Chart: Bd21 #07
| Category | Bd21 #07_F(t) | Bd21 #07 | Bd21 #07_Fler(t) | Bd21 #07 LER calc |
|---|---|---|---|---|
### Chart: Bd21 #06
| Category | Bd21 #06_F(t) | Bd21 #06 | Bd21 #06_Fler(t) | Bd21 #06 LER calc |
|---|---|---|---|---|
### Chart: Bd21 #08
| Category | Bd21 #08_F(t) | Bd21 #08 | Bd21 #08_Fler(t) | Bd21 #08 LER calc |
|---|---|---|---|---|
### Chart: Bd21 #09
| Category | Bd21 #09_F(t) | Bd21 #09 | Bd21 #09_Fler(t) | Bd21 #09 LER calc |
|---|---|---|---|---|

## Slide 7
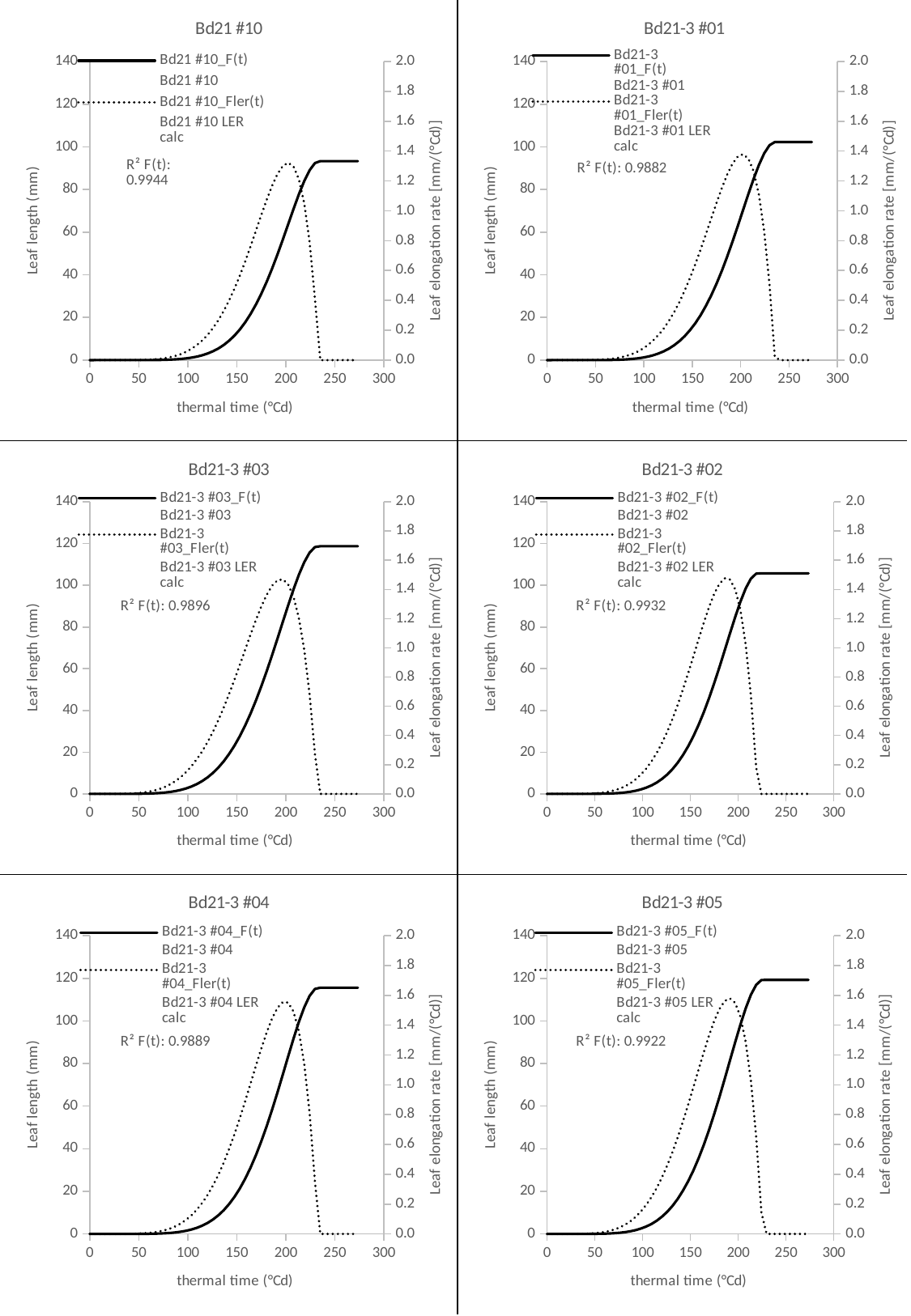

### Chart: Bd21-3 #01
| Category | Bd21-3 #01_F(t) | Bd21-3 #01 | Bd21-3 #01_Fler(t) | Bd21-3 #01 LER calc |
|---|---|---|---|---|
### Chart: Bd21 #10
| Category | Bd21 #10_F(t) | Bd21 #10 | Bd21 #10_Fler(t) | Bd21 #10 LER calc |
|---|---|---|---|---|
### Chart: Bd21-3 #03
| Category | Bd21-3 #03_F(t) | Bd21-3 #03 | Bd21-3 #03_Fler(t) | Bd21-3 #03 LER calc |
|---|---|---|---|---|
### Chart: Bd21-3 #02
| Category | Bd21-3 #02_F(t) | Bd21-3 #02 | Bd21-3 #02_Fler(t) | Bd21-3 #02 LER calc |
|---|---|---|---|---|
### Chart: Bd21-3 #04
| Category | Bd21-3 #04_F(t) | Bd21-3 #04 | Bd21-3 #04_Fler(t) | Bd21-3 #04 LER calc |
|---|---|---|---|---|
### Chart: Bd21-3 #05
| Category | Bd21-3 #05_F(t) | Bd21-3 #05 | Bd21-3 #05_Fler(t) | Bd21-3 #05 LER calc |
|---|---|---|---|---|

## Slide 8
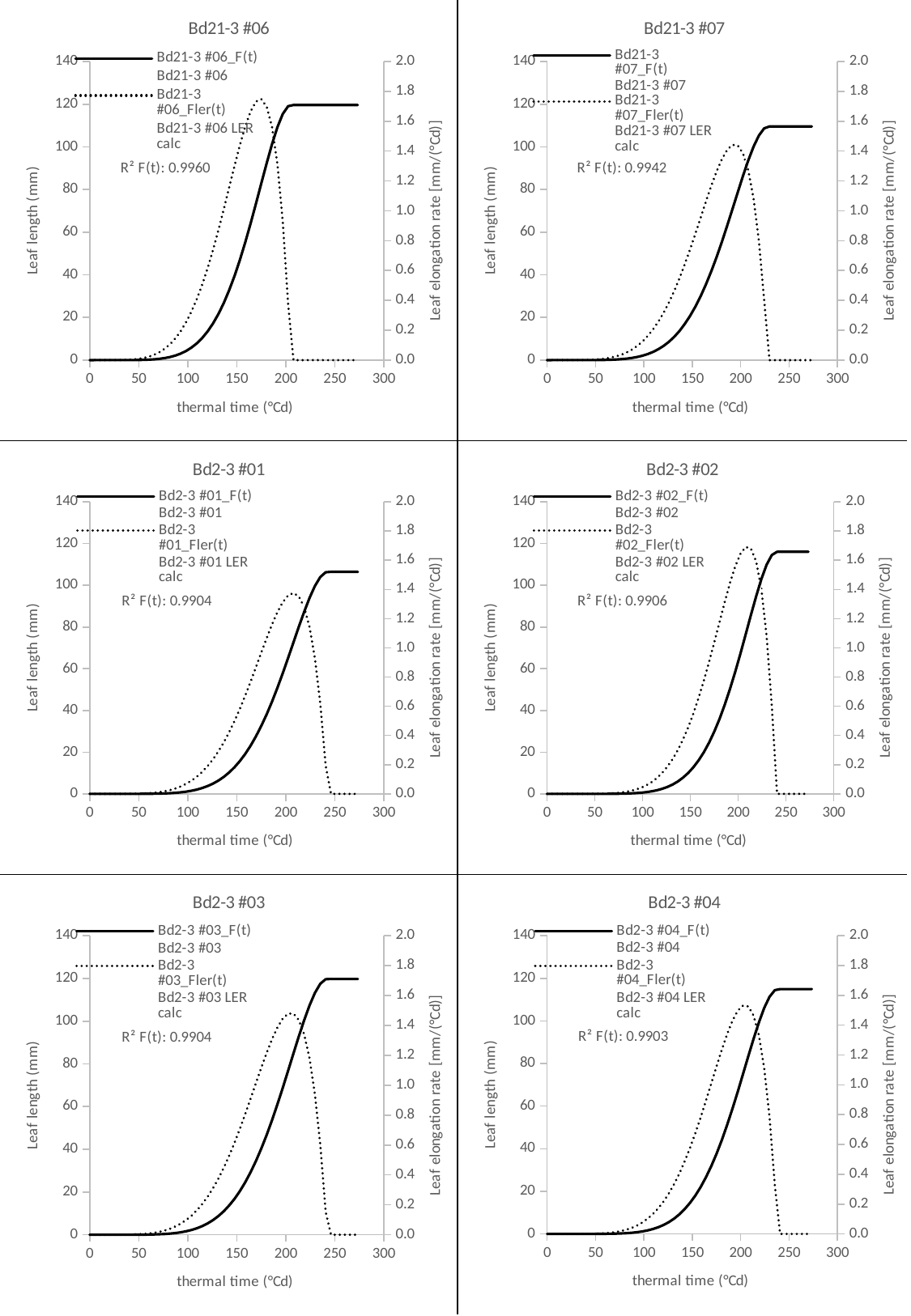

### Chart: Bd21-3 #06
| Category | Bd21-3 #06_F(t) | Bd21-3 #06 | Bd21-3 #06_Fler(t) | Bd21-3 #06 LER calc |
|---|---|---|---|---|
### Chart: Bd21-3 #07
| Category | Bd21-3 #07_F(t) | Bd21-3 #07 | Bd21-3 #07_Fler(t) | Bd21-3 #07 LER calc |
|---|---|---|---|---|
### Chart: Bd2-3 #01
| Category | Bd2-3 #01_F(t) | Bd2-3 #01 | Bd2-3 #01_Fler(t) | Bd2-3 #01 LER calc |
|---|---|---|---|---|
### Chart: Bd2-3 #02
| Category | Bd2-3 #02_F(t) | Bd2-3 #02 | Bd2-3 #02_Fler(t) | Bd2-3 #02 LER calc |
|---|---|---|---|---|
### Chart: Bd2-3 #03
| Category | Bd2-3 #03_F(t) | Bd2-3 #03 | Bd2-3 #03_Fler(t) | Bd2-3 #03 LER calc |
|---|---|---|---|---|
### Chart: Bd2-3 #04
| Category | Bd2-3 #04_F(t) | Bd2-3 #04 | Bd2-3 #04_Fler(t) | Bd2-3 #04 LER calc |
|---|---|---|---|---|

## Slide 9
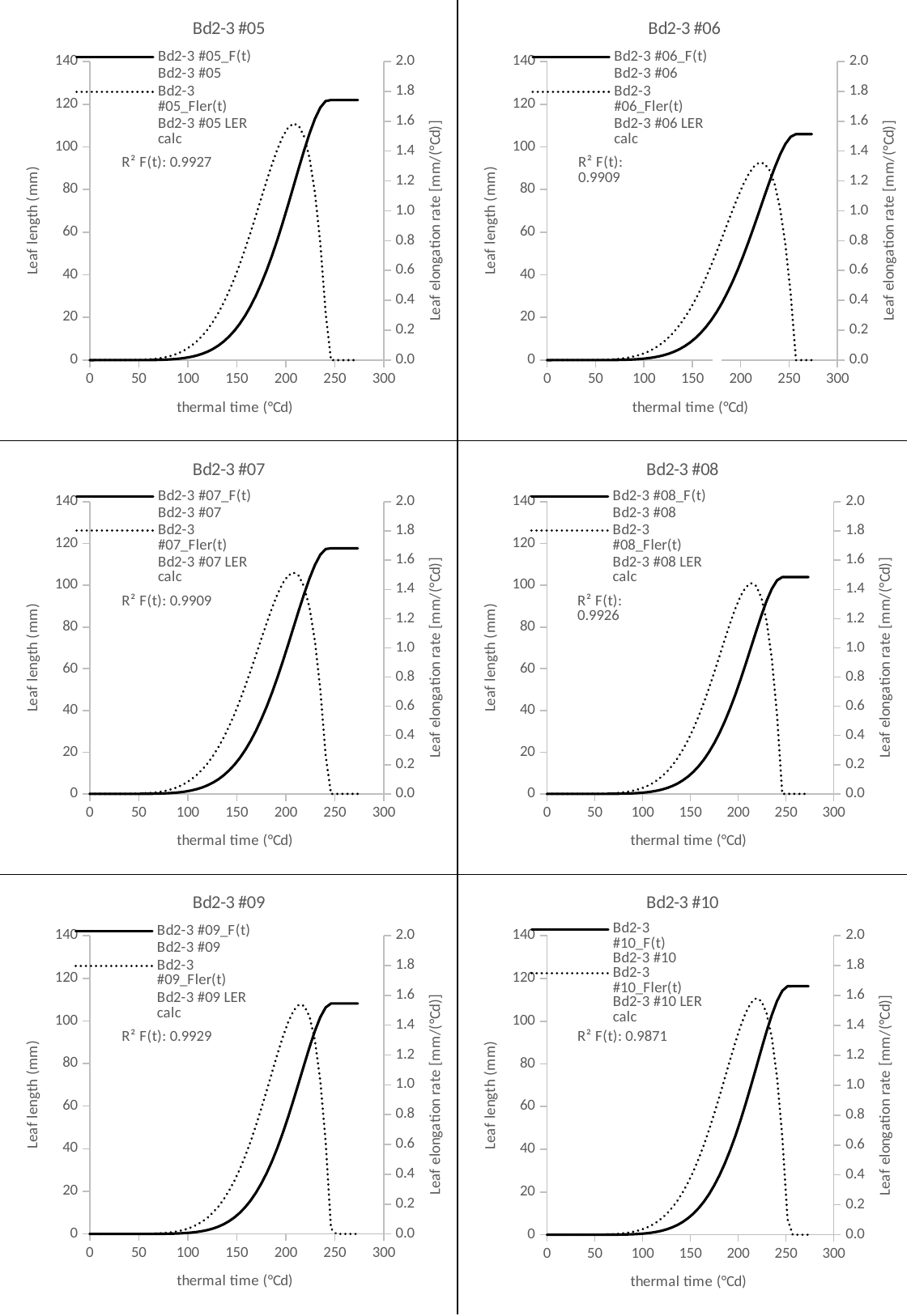

### Chart: Bd2-3 #05
| Category | Bd2-3 #05_F(t) | Bd2-3 #05 | Bd2-3 #05_Fler(t) | Bd2-3 #05 LER calc |
|---|---|---|---|---|
### Chart: Bd2-3 #06
| Category | Bd2-3 #06_F(t) | Bd2-3 #06 | Bd2-3 #06_Fler(t) | Bd2-3 #06 LER calc |
|---|---|---|---|---|
### Chart: Bd2-3 #07
| Category | Bd2-3 #07_F(t) | Bd2-3 #07 | Bd2-3 #07_Fler(t) | Bd2-3 #07 LER calc |
|---|---|---|---|---|
### Chart: Bd2-3 #08
| Category | Bd2-3 #08_F(t) | Bd2-3 #08 | Bd2-3 #08_Fler(t) | Bd2-3 #08 LER calc |
|---|---|---|---|---|
### Chart: Bd2-3 #09
| Category | Bd2-3 #09_F(t) | Bd2-3 #09 | Bd2-3 #09_Fler(t) | Bd2-3 #09 LER calc |
|---|---|---|---|---|
### Chart: Bd2-3 #10
| Category | Bd2-3 #10_F(t) | Bd2-3 #10 | Bd2-3 #10_Fler(t) | Bd2-3 #10 LER calc |
|---|---|---|---|---|

## Slide 10
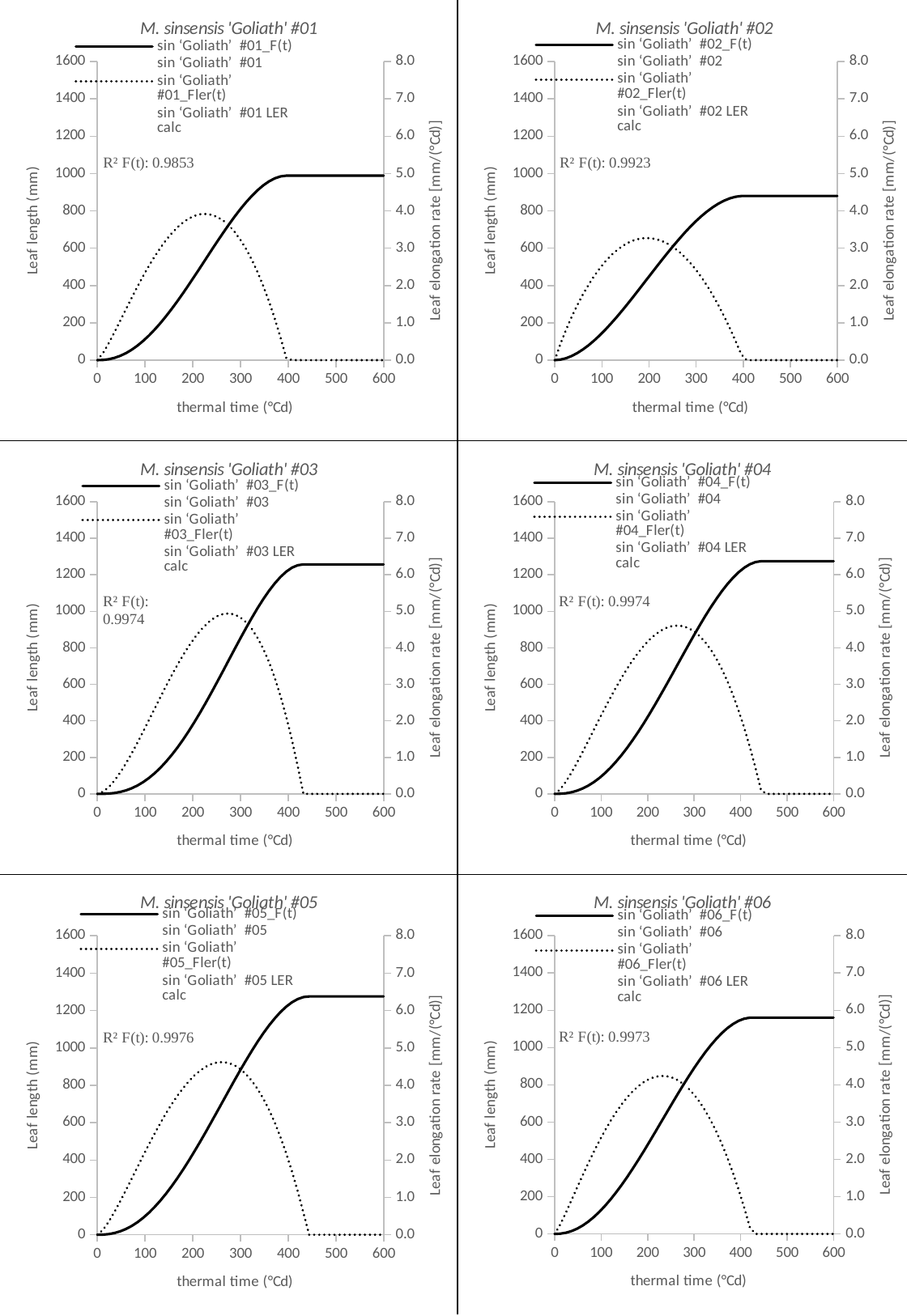

### Chart: M. sinsensis 'Goliath' #01
| Category | sin ‘Goliath’ #01_F(t) | sin ‘Goliath’ #01 | sin ‘Goliath’ #01_Fler(t) | sin ‘Goliath’ #01 LER calc |
|---|---|---|---|---|
### Chart: M. sinsensis 'Goliath' #02
| Category | sin ‘Goliath’ #02_F(t) | sin ‘Goliath’ #02 | sin ‘Goliath’ #02_Fler(t) | sin ‘Goliath’ #02 LER calc |
|---|---|---|---|---|
### Chart: M. sinsensis 'Goliath' #03
| Category | sin ‘Goliath’ #03_F(t) | sin ‘Goliath’ #03 | sin ‘Goliath’ #03_Fler(t) | sin ‘Goliath’ #03 LER calc |
|---|---|---|---|---|
### Chart: M. sinsensis 'Goliath' #04
| Category | sin ‘Goliath’ #04_F(t) | sin ‘Goliath’ #04 | sin ‘Goliath’ #04_Fler(t) | sin ‘Goliath’ #04 LER calc |
|---|---|---|---|---|
### Chart: M. sinsensis 'Goliath' #05
| Category | sin ‘Goliath’ #05_F(t) | sin ‘Goliath’ #05 | sin ‘Goliath’ #05_Fler(t) | sin ‘Goliath’ #05 LER calc |
|---|---|---|---|---|
### Chart: M. sinsensis 'Goliath' #06
| Category | sin ‘Goliath’ #06_F(t) | sin ‘Goliath’ #06 | sin ‘Goliath’ #06_Fler(t) | sin ‘Goliath’ #06 LER calc |
|---|---|---|---|---|

## Slide 11
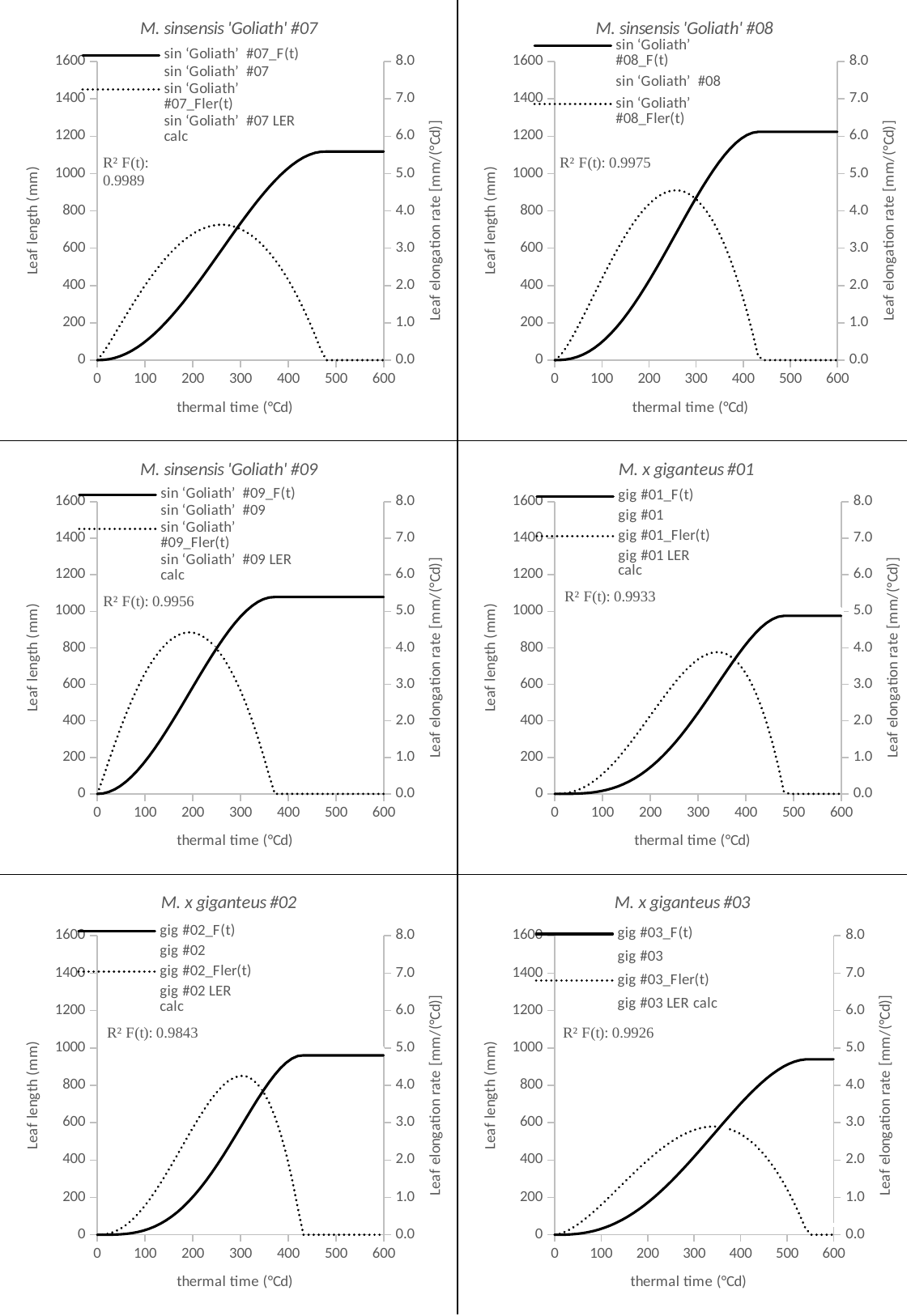

### Chart: M. sinsensis 'Goliath' #07
| Category | sin ‘Goliath’ #07_F(t) | sin ‘Goliath’ #07 | sin ‘Goliath’ #07_Fler(t) | sin ‘Goliath’ #07 LER calc |
|---|---|---|---|---|
### Chart: M. sinsensis 'Goliath' #08
| Category | sin ‘Goliath’ #08_F(t) | sin ‘Goliath’ #08 | sin ‘Goliath’ #08_Fler(t) | sin ‘Goliath’ #08 LER calc |
|---|---|---|---|---|
### Chart: M. sinsensis 'Goliath' #09
| Category | sin ‘Goliath’ #09_F(t) | sin ‘Goliath’ #09 | sin ‘Goliath’ #09_Fler(t) | sin ‘Goliath’ #09 LER calc |
|---|---|---|---|---|
### Chart: M. x giganteus #01
| Category | gig #01_F(t) | gig #01 | gig #01_Fler(t) | gig #01 LER calc |
|---|---|---|---|---|
### Chart: M. x giganteus #02
| Category | gig #02_F(t) | gig #02 | gig #02_Fler(t) | gig #02 LER calc |
|---|---|---|---|---|
### Chart: M. x giganteus #03
| Category | gig #03_F(t) | gig #03 | gig #03_Fler(t) | gig #03 LER calc |
|---|---|---|---|---|

## Slide 12
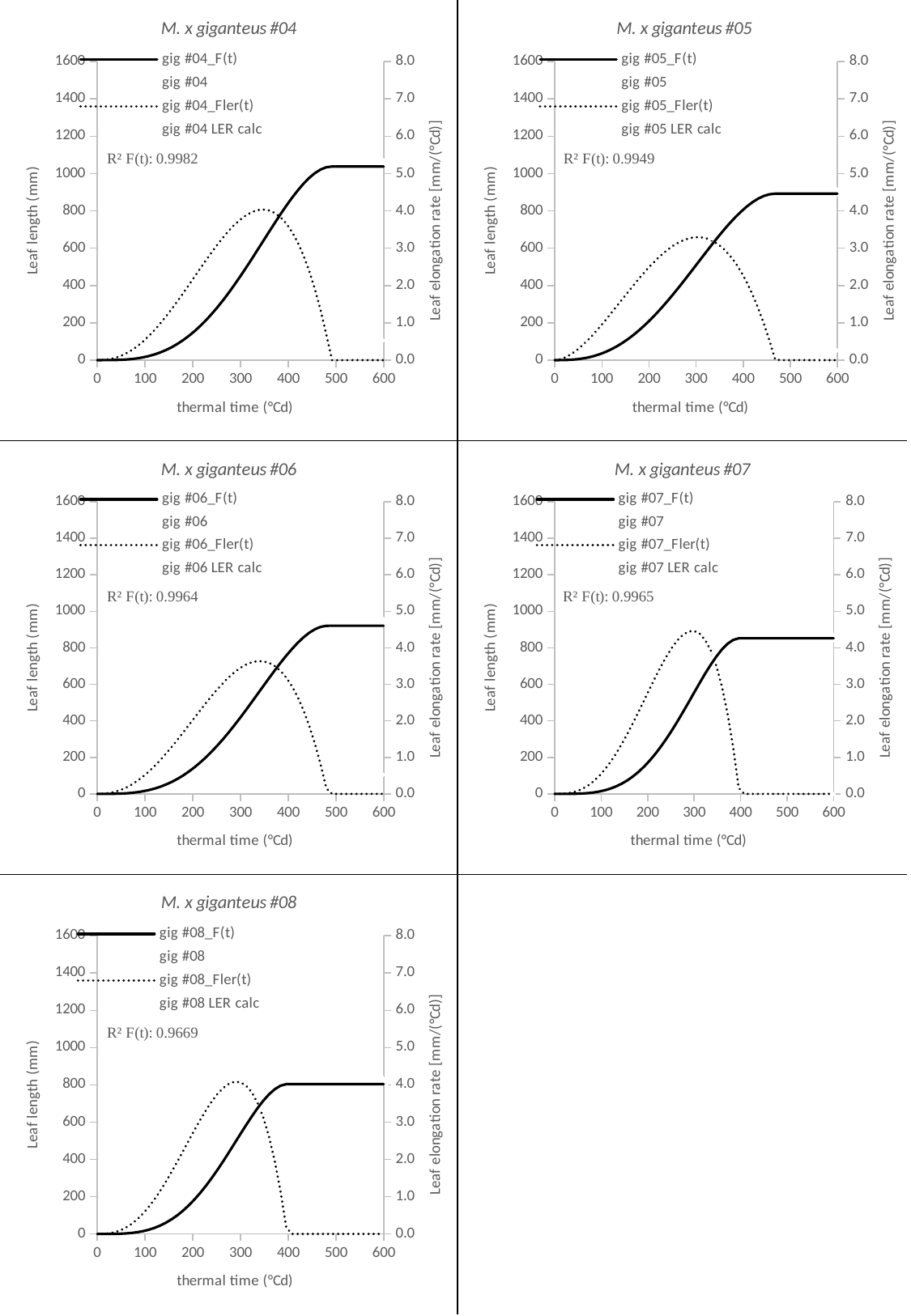

### Chart: M. x giganteus #04
| Category | gig #04_F(t) | gig #04 | gig #04_Fler(t) | gig #04 LER calc |
|---|---|---|---|---|
### Chart: M. x giganteus #05
| Category | gig #05_F(t) | gig #05 | gig #05_Fler(t) | gig #05 LER calc |
|---|---|---|---|---|
### Chart: M. x giganteus #06
| Category | gig #06_F(t) | gig #06 | gig #06_Fler(t) | gig #06 LER calc |
|---|---|---|---|---|
### Chart: M. x giganteus #07
| Category | gig #07_F(t) | gig #07 | gig #07_Fler(t) | gig #07 LER calc |
|---|---|---|---|---|
### Chart: M. x giganteus #08
| Category | gig #08_F(t) | gig #08 | gig #08_Fler(t) | gig #08 LER calc |
|---|---|---|---|---|
