# Supplementary figures and images for: LEAF-E: a tool to analyze grass leaf growth using function fitting
Source: Plant Methods. 2014 Nov 6;10:37. doi: 10.1186/1746-4811-10-37 (PMC4246515; doi:10.1186/1746-4811-10-37)

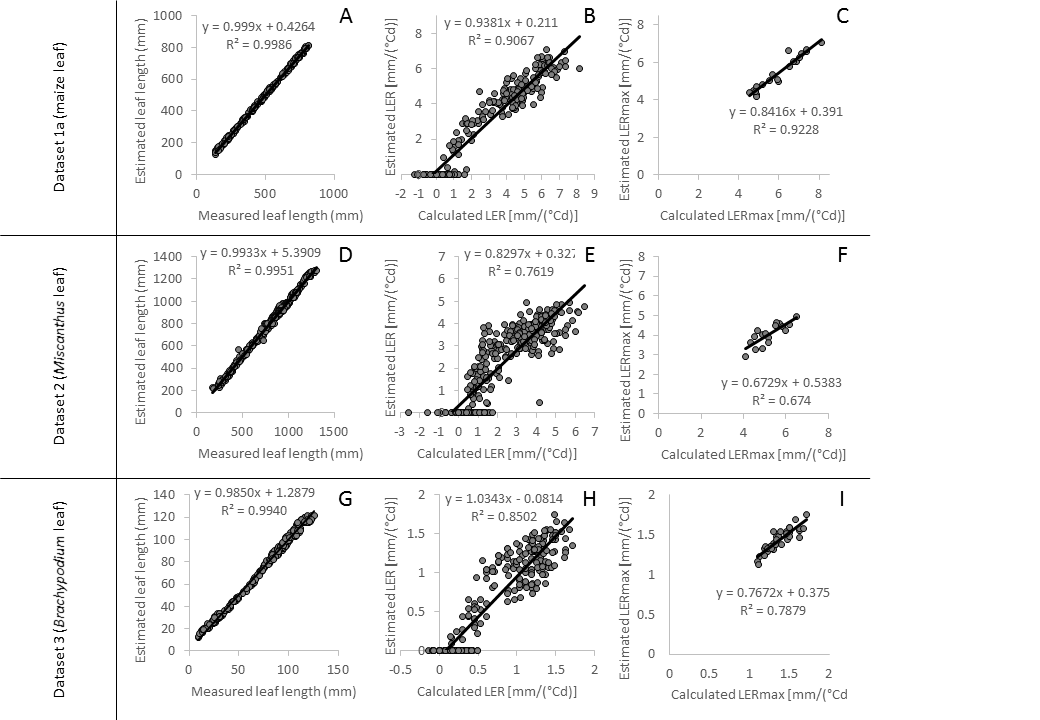

Supplement: Supplementary file 2 — Additional file 2: Scatter plots and linear regression for datasets of leaf length measurements. Plots show estimated to measured leaf lengths (A, D, G), estimated LER to calculated LER (calculated as leaf length increase between two consecutive measurements divided by the respective thermal time interval) (B, E, H) and estimated LERmax to LERmax determined as maximal value of the profile of calculated LER (C, F, I), all on a plant-by-plant basis, for datasets of maize (dataset 1a: A, B, C), Miscanthus (dataset 2: D, E, F) and Brachypodium (dataset 3: G, H, I). (TIFF 213 KB) [file 13007_2014_305_MOESM2_ESM.tiff]

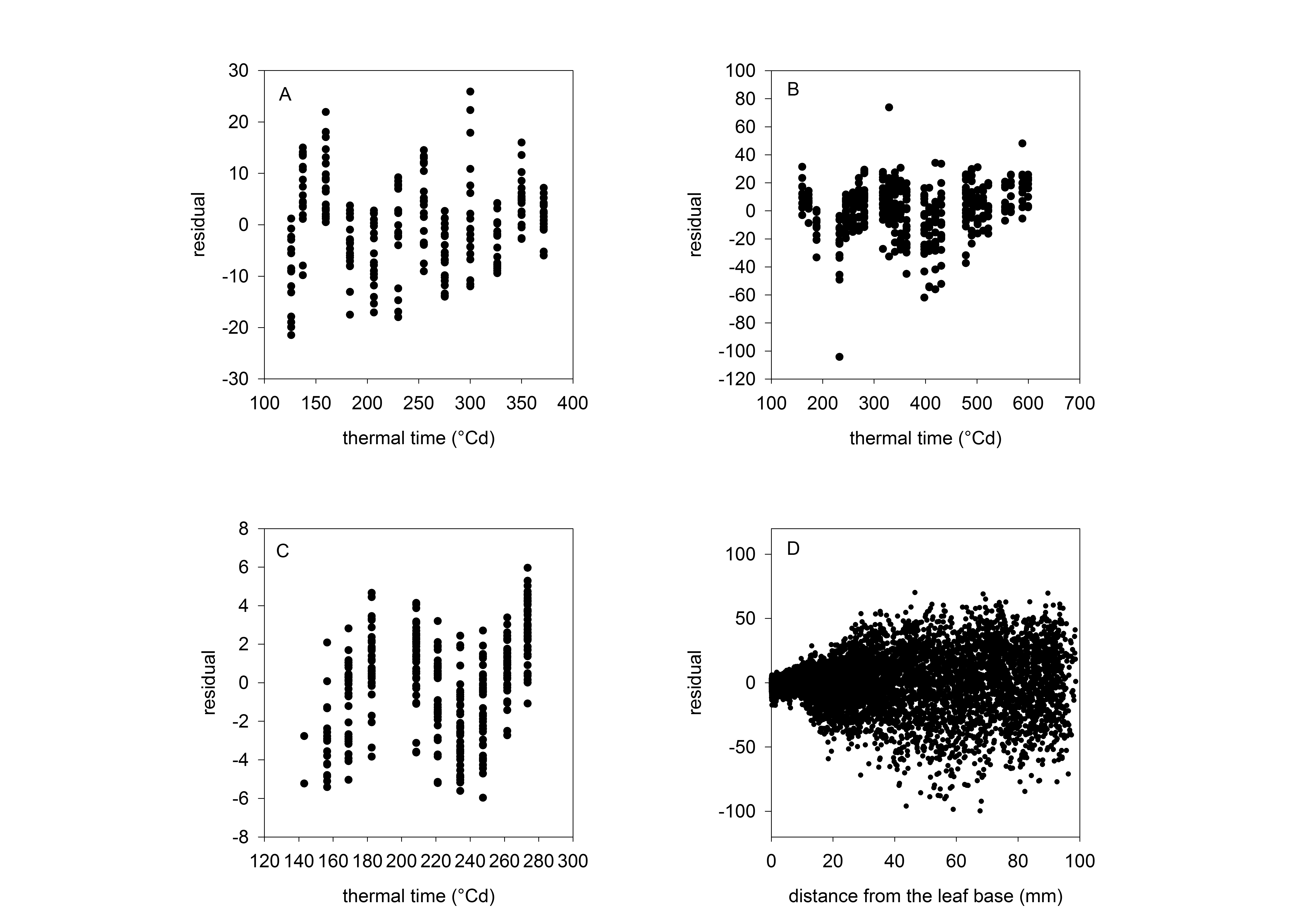

Supplement: Supplementary file 3 — Additional file 3: Distribution of the residuals for the fitting of the beta sigmoid function to leaf length data of maize (A), Miscanthus spp. (B) and Brachypodium spp. (C) and cell length data of maize (D) using LEAF-E. (JPEG 2 MB) [file 13007_2014_305_MOESM3_ESM.jpeg]

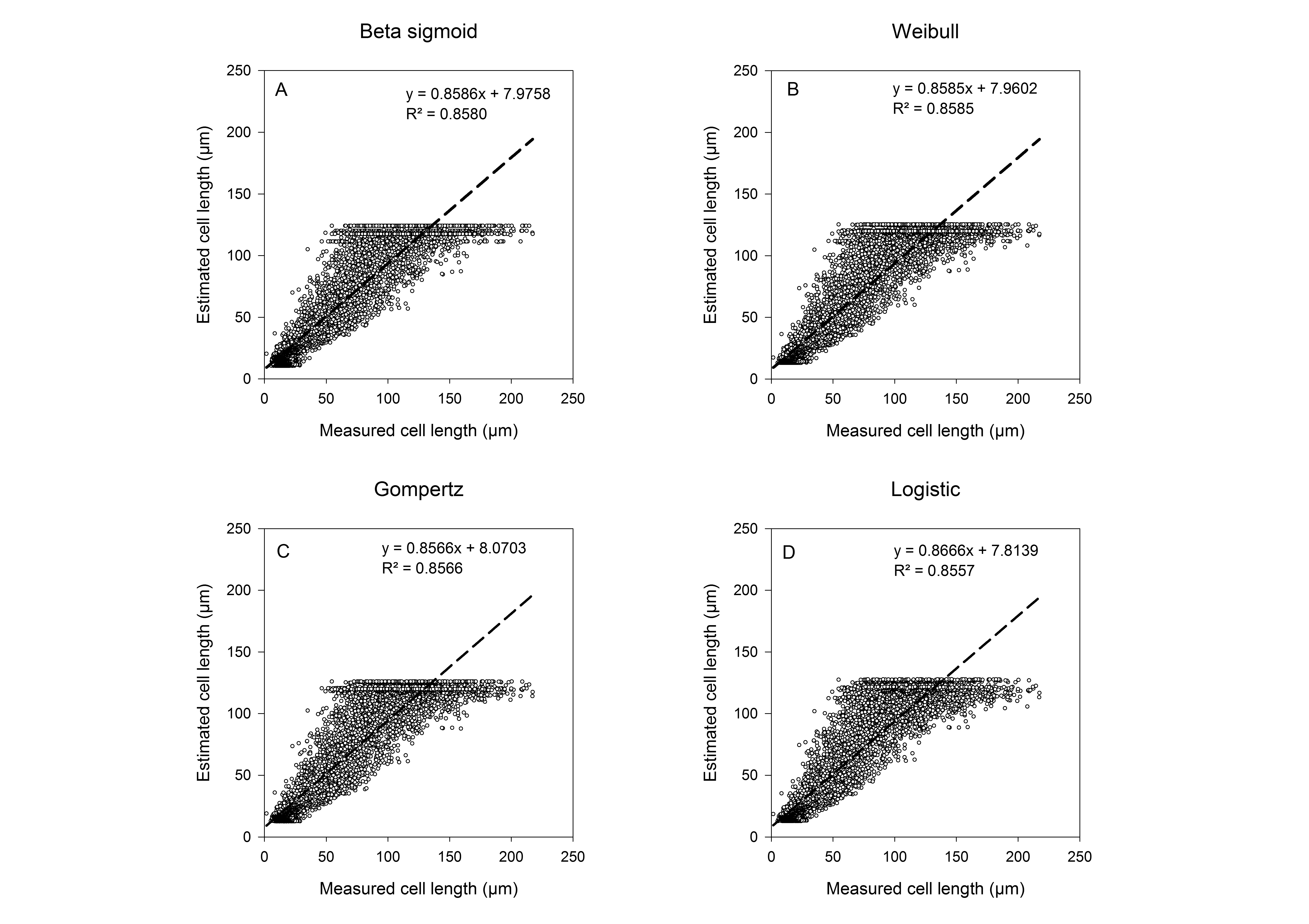

Supplement: Supplementary file 6 — Additional file 6: Comparison of goodness of fit of the Beta sigmoid (A), Weibull (B), Gompertz (C) and Logistic (D) functions for the cell length profile of the 4 th leaf in maize. The plots show the linear regression of estimated versus measured cell lengths of all data points of dataset 1b. (JPEG 3 MB) [file 13007_2014_305_MOESM6_ESM.jpeg]
